# The safety and efficacy of neutral electrolyzed water solution for wound irrigation: post-market clinical follow-up study

**DOI:** 10.3389/fdsfr.2024.1402684

**Published:** 2025-01-16

**Authors:** Veronika Valdová, Vladimíra Štěpánová, Lenka Lapčíková

**Affiliations:** ^1^ Independent Consultant, Prague, Czechia; ^2^ NewWaterMeaning, s.r.o., Prague, Czechia

**Keywords:** neutral electrolyzed water, superoxidized solution, wound irrigation, chronic wound, hypochlorous acid, venous leg ulcer, diabetic foot, pressure ulcer

## Abstract

**Introduction:**

Chronic wounds are a significant public health challenge, representing a considerable burden on the healthcare system. There are numerous gaps in knowledge in the treatment of chronic wounds. First, it is difficult to follow patients through different types of care. Wounds in polymorbid, elderly patients often remain unhealed due to the patient succumbing to their primary disease. No reliable data exist regarding the time to wound closure, type of interventions, the use of antibiotics, the nature and rate of complications, or the causes of treatment failures.

**Methods:**

This Post-Market Clinical Follow-Up (PMCF) study is a prospective, multicentric, observational, descriptive, qualitative survey among healthcare professionals that involves 237 patients with acute and chronic wounds treated with superoxide-based wound irrigation solution DebriEcaSan Alfa in real-world settings over 12 weeks, both outpatient and inpatient. The study aimed to collect additional clinical data to confirm the safety, performance, and clinical benefit of DebriEcaSan Alfa.

**Results:**

The Manufacturer collected 237 survey forms from 81 healthcare facilities, nursing homes, and outpatient clinics in the Czech Republic. The most common diagnoses were venous leg ulcer, pressure ulcer, diabetic foot ulcer, and traumatic wound. The most common comorbidities and risk factors were obesity, diabetes mellitus, and peripheral artery disease. Significant improvement was observed in all parameters, including pain, malodor, affected tissues, reduction in wound size, and granulation and epithelization. A marked reduction in size was observed in all wound size categories. 19 (8%) patients healed by end of week 6; and 66 (28%) healed by week 9. 130 (55%) patients were considered healed by week 12.

**Discussion:**

The current clinical practice guidelines refrain from recommending any of the available irrigation solutions and wound dressings due to low-quality evidence. Superoxidized solutions have excellent biocompatibility and are non-cytotoxic, non-sensitizing, not irritating, non-genotoxic, and have broad-spectrum antimicrobial properties. There is no objective baseline to compare the results to, as typical healing times in a comparable population are not accessible. No single standard of care exists in the treatment of chronic wounds, and significant variability in practices exists across the health system.

## 1 Introduction

Chronic wounds are a significant public health challenge, representing a considerable burden on healthcare systems. The estimated prevalence of chronic wounds is 2.21 per 1,000 population, the majority of which are chronic leg ulcers (Martinengo et al., 2019). The most common types of chronic wounds are diabetic foot ulcers, venous leg ulcers, and pressure ulcers. In diabetic patients, the annual risk of foot ulceration is around 2%, whereas the lifetime risk is 12%–25% (Nagoba et al., 2021). The prevalence of leg ulcers is estimated to range from 0.045% to 1.5% in the United Kingdom (Chaplin, 2020) and 0.08% in Germany (Rüttermann et al., 2013). The prevalence of pressure ulcers in inpatient settings approximates 22% (Dreifke et al., 2015). In Germany, a significant cost of inpatient medical care is spent on treating venous leg ulcers and diabetic foot ulcers (Rüttermann et al., 2013). In the Czech Republic, the incidence and prevalence of chronic wounds and leg ulcers, in particular, follow the trends in other developed countries. The prevalence of patients with diabetes increased from 78 per 1,000 people in 2007 to 88 per 1,000 people in 2017 (UZIS, 2018). Around 4% of diabetics develop diabetic foot syndrome, of which 24% result in amputation (Jirkovská, 2018). Despite the significant impact on the health system and patients’ quality of life, treating chronic wounds remains an under-researched area.

Clinical management of chronic wounds relies on aggressive debridement and exudate and moisture management to facilitate granulation and epithelization to achieve wound closure. There is a consensus that effective debridement, reducing bioburden, and infection control are the cornerstones of the treatment of chronic wounds (Eriksson et al., 2022). Additional interventions include the management of systemic diseases such as diabetes, compression in venous leg ulcers, restoration of arterial inflow in ischemic ulcers, and offloading in diabetic foot ulcers (Schultz et al., 2003). Evidence-based recommendations for patients with infected diabetic foot favor hydrogel and hyperbaric oxygenation (Rüttermann et al., 2013) and advise against the use of medicinal honey, growth factors, silver preparations, bacteriophage therapy, or negative-pressure wound therapy, and antiseptics in general. However, this recommendation is conditional, and the certainty of the evidence is low (Lazzarini et al., 2023; Senneville et al., 2024). Venous leg ulcers are typically treated with compression bandages, debridement, and irrigation with normal saline, water, or antiseptics. Insufficient evidence from randomized clinical trials exists to recommend optimal approaches to cleansing venous leg ulcers (McLain et al., 2021).

A significant variability in wound care practices exists across the health system. A wide variety of products available on the market are empirically used in a variety of clinical contexts, a multitude of deployment methods, and countless combinations. Jones et al. (2007) studied the consistency of current chronic wound care practices in the U.S. and found significant variations in adherence across sites of care delivery (Jones et al., 2007). This lack of consistency makes it very difficult to compare data across facilities. No reliable data exists regarding the time to wound closure, the type of interventions and the sequence and duration of their use, the use of antibiotics, the nature and rate of complications, or the causes of treatment failures. Additionally, very few quality studies focus on the treatment of chronic wounds. Consequently, low-quality evidence results in low-confidence recommendations in clinical guidelines (Eriksson et al., 2022). This lack of evidence further exacerbates the existing problem with the variability of treatment approaches across health systems.

Inaccurate or sporadic reporting does not allow adequate use of data to monitor treatment outcomes. As documented by Pokorná et al. (2017) in her study on cutaneous ulcer diseases and their reporting in acute inpatient care in the Czech Republic, the typical healing times for specific types of ulcers are not easily obtainable from medical records, and no reliable benchmarks currently exist. Pokorná examined data from the National Register of Hospitalized Persons (NRHOSP) and Death Examination Reports from 2007 to 2015 as part of project DRG Restart. She stressed the issue of underreporting hospital-acquired ulcers, and the limited value of incomplete data obtained from the National Health Information System and reference hospitals, making it impossible to calculate the burden of hospitalizations involving chronic wounds. Consequently, it is difficult to consider the impact of ulcer diseases and the cost of treatment across the board (Pokorná et al., 2017).

Moreover, treatment outcomes such as quality of healing, complications, quality of life, burden on healthcare staff, and affordability are inconsistently used across studies and in quality-of-care metrics, making the results difficult to compare (Eriksson et al., 2022). Endpoints for chronic cutaneous ulcer studies are the time to healing, wound size reduction, infection control, the need for amputation, prevention of recurrence, improved functionality, and reduced isolation (Eaglstein et al., 2012). While time to wound closure and wound size reduction are the primary outcomes, a healed wound is not always the expected outcome. In palliative wound care, the desired outcomes include pain and malodor reduction, exudate management, and other quality-of-life measures (Eriksson et al., 2022).

Amputation is an important complication of infected chronic wounds and, especially, diabetic foot ulcers. High amputations are the consequence of late hospitalizations, deep defect or phlegmon, Charcot osteoarthropathy, insufficiently treated infection, severe ischemia of the lower limbs, poorly controlled diabetes, smoking, atherosclerosis, and renal insufficiency. The acceleration of atherosclerosis risk factors after amputation leads to the worsening of cardiovascular diseases, persistent neuropathy complications on the stump of the amputated limb, and premature death (Jirkovská, 2018). Jirkovská’s findings raise important points about the advanced condition often observed at the initial examination regarding wound characteristics, which, combined with patient comorbidities and risk factors, adversely impact treatment outcomes.

Numerous gaps in knowledge exist in treating chronic wounds. While consensus exists about the importance of wound cleansing and debridement (Dayya et al., 2022; Eriksson et al., 2022), no clear recommendations are available regarding the optimal choice and method of use of wound irrigation for diabetic foot (Senneville et al., 2024), infected leg ulcers (National Institute for Health and Care Excellence, 2020) or pressure ulcers (European Pressure Ulcer Advisory Panel, National Pressure Injury Advisory Panel and Pan Pacific Pressure Injury Alliance, 2019).

The evidence regarding best wound irrigation practices is sparse, and no official recommendations currently exist from any healthcare organization (Saeg et al., 2021). Irrigation practices vary widely in terms of delivery method, volume, and type of solution. The majority of wound irrigation solutions are cytotoxic, and their efficacy to enhance healing is uncertain (Wilkins and Unverdorben, 2013). Comprehensive systematic reviews by Alonso-Coello et al. (2016b) did not identify any direct evidence to support the use of any specific wound irrigation solutions or wound cleansing techniques. An ideal irrigation solution should be isotonic, nonhemolytic, noncytotoxic, transparent, easy to sterilize, and inexpensive. The ideal antiseptic solution is still debated, although the current literature favors the use of normal saline for non-infected wounds (Gabriel, 2021). The European Pressure Ulcer Advisory Panel (EPUAP) guideline from 2019 recommends the use of antimicrobial solutions to clean pressure injuries with suspected or confirmed infection, such as polyhexamethylene biguanide (PHMB), octenidine dihydrochloride (OCT), superoxidized solution with hypochlorous acid (HOCL) and sodium hypochlorite (NaOCL), and povidone iodine, rather than normal saline, sterile water, or potable tap water. However, the recommendations are based only on expert opinions (European Pressure Ulcer Advisory Panel, National Pressure Injury Advisory Panel and Pan Pacific Pressure Injury Alliance, 2019). Other guidelines suggest that topical antiseptics or antimicrobials shall not be routinely used to treat diabetic and pressure ulcers (National Institute for Health and Care Excellence, 2016). No specific advice on wound irrigation methods is given in the 2023 IWGDF Guidelines on the prevention and management of diabetes-related foot disease (Senneville et al., 2024).

The low confidence recommendations stem from the low quality of evidence from wound care studies. As Forster and Pagnamenta (2015) noted in their Cochrane review, designing randomized controlled trials (RCTs) in wound care is challenging due to the significant variability of patient demographics, wound characteristics, comorbidities, and risk factors as well as concurrent therapies and self-care. Therefore, recruiting enough participants with comparable characteristics represents a major challenge. Common limitations of RCTs in wound care include poor baseline characteristics, sample sizes too low to reliably detect differences between treatments, poor reporting of assessor blinding, randomization methods and allocation concealment, and inadequate follow-up. Important endpoints such as pain, malodor, frequency of dressing changes, patient satisfaction, study withdrawals, and adverse events are often not reported. Inappropriate comparators can limit the generalizability of the results to a real-world population. The overall quality of clinical evidence is suboptimal and insufficient to inform clinical practice (Forster and Pagnamenta, 2015).

The organization of healthcare that serves wound care patients produces additional challenges. Most clinical data available in scientific literature and national registries come from hospitalized patients rather than outpatient care. As stated by Pokorná et al. (2017), it is impossible to follow patients through their transition through different types of care, i.e., from the first occurrence of the wound and the first contact with a healthcare professional to outpatient treatment, hospitalization, discharge with or without home care assistance or transfer to a long-term care facility or a nursing home. Each of these care modalities has its own treatment protocols and methods of measuring treatment outcome. Since many chronic wounds develop in polymorbid, elderly patients as a complication of their underlying disease, wounds often remain unhealed due to the patient succumbing to their primary disease. The time to wound closure of complex, non-healing wounds cannot be currently obtained from data gathered within the existing quality management systems. The only obtainable data point is time to discharge, meaning the wound is manageable in an outpatient setting or with the assistance of a homecare nursing service. Similarly, no reliable data from the existing monitoring systems details the type of interventions, the use of antibiotics, the nature and rate of complications, or the causes of treatment failures (Pokorná et al., 2017).

Hence, the present study has been conducted to determine the efficacy and safety of superoxidized solution (DebriEcaSan Alfa) in the treatment of chronic wounds. This Post-Market clinical Follow-Up (PMCF) study is a prospective, multicentric, observational, descriptive, qualitative survey among healthcare professionals. The study involves 237 patients with acute and chronic wounds who were treated with superoxide-based wound irrigation solution DebriEcaSan Alfa (NewWaterMeaning s.r.o.) in real-world settings. The PMCF meets the requirements outlined in the EU Regulation 2017/745 on medical devices. A literature review was performed to update current knowledge about superoxide-based wound irrigation solutions, their antimicrobial and antibiofilm properties, and the state of the art in treating chronic wounds.

## 2 Materials and methods

### 2.1 Type of study

This is a post-market clinical follow-up (PMCF) study sponsored and performed by the Manufacturer of DebriEcaSan Alfa, NewWaterMeaning, s.r.o., Czech Republic. Since the product was used within its intended purpose, and the patients were not submitted to invasive or burdensome procedures additional to those performed under the normal conditions of use of the device, no approval of ethics committee was required (Jurrmann, 2023). The study is part of the Manufacturer’s Post-Market Surveillance Plan and it is conducted in compliance with European Medical Device Regulation (EU MDR) 2017/745 (European Commission, 2017).

### 2.2 Study design

This PMCF is a prospective, multicentric, observational, descriptive, qualitative survey among healthcare professionals that involves 237 patients treated with DebriEcaSan Alfa in real-world settings, both outpatient and inpatient.

### 2.3 Eligible subjects

Patients of all demographics with acute or chronic wounds of any origin and any duration who were treated with DebriEcaSan Alfa as part of their standard protocol in outpatient and inpatient facilities or nursing homes in the Czech Republic were considered.

### 2.4 Main outcomes

The study shall provide insight into the characteristics of treated population in terms of age, sex, comorbidities, and risk factors, and the initial wound characteristics in real world settings. The data shall establish baseline in terms of expected healing times and complication rates for different types of chronic wounds, especially wounds that are large, deep, and infected, and wounds in patients with multiple comorbid conditions. Important outcomes are the reduction of wound size, malodor, pain, patient’s comfort, and ease of use for healthcare personnel.

### 2.5 PMCF plan

The survey form enquires about the patient’s demographics, basic diagnosis, comorbidities and risk factors, characteristics of the wound at initial examination, and at weeks 3, 6, 9, and 12, methods of use of DebriEcaSan Alfa, treatment outcome, complications, commentary, consent and signature. Case studies are supported by imagery that documents the healing process (where patient consent to share images exists). Data was collected from healthcare professionals using a survey form. The current dataset concerns survey forms collected between January 2019 and December 2023. The study is still ongoing.

### 2.6 Assessment

The data collected from healthcare professionals through survey forms was compiled in an Excel spreadsheet and presented in a series of graphs and tables. Each component was evaluated in the context of existing evidence, including information held by the Manufacturer and data from scientific literature.

### 2.7 Review of literature

The Manufacturer regularly screens databases PubMed, Prospero, Cochrane Database of Systematic Reviews, NICE guidelines, and ClinicalTrials.gov for publications as part of its post-market surveillance activities to update its technical documentation, specifically Biological Compatibility Assessment, Clinical Evaluation, and Post-market Clinical Follow-Up for superoxidized solutions and gels. An overview of accumulated knowledge is provided along with an update for the monitoring period from 1 October 2021 to 30 September 2023.

### 2.8 Eligibility criteria

Articles focusing on the use superoxidized solutions in wound irrigation, their antimicrobial efficacy and cytotoxicity were sought. *In vivo* and *in vitro* studies were considered to evaluate the cytotoxicity and antimicrobial properties of superoxidized solutions. Clinical practice guidelines, systematic reviews and meta-analyses, and focused review articles were screened to establish the current standard of care and state-of-the-art in the treatment of chronic and non-healing wounds. Clinical studies comparing different irrigation and antiseptic solutions in the treatment of acute and chronic wounds were examined to review the safety and efficacy of superoxidized solutions in the context of other available products in different clinical scenarios. Articles published in selected scientific electronic databases from 1 October 2021 to 30 September 2023 were considered.

### 2.9 Exclusion criteria

Publications that did not discuss a relevant device and purpose of use, studies which did not focus on the use of superoxidized solutions in wound care, those published outside of the indicated period, and publications that do not contribute to the state of the art were excluded.

### 2.10 Search strategy

Electronic scientific databases PubMed, Prospero, Cochrane Database of Systematic Reviews, NICE guidelines, and ClinicalTrials.gov were electronically searched and subsequently hand searched to retrieve relevant sources. English was chosen as the search language. The search strategy was implemented via the following steps: an initial search was performed using the keywords: “superoxidized solution,” OR “neutral electrolyzed water,” OR “hypochlorous acid,” AND “chronic wound,” OR “diabetic foot ulcer” OR “venous leg ulcer” OR “pressure ulcer” in electronic databases. The results of the initial search were combined into a single set. Duplicities were removed and then the titles, abstracts and full texts of the obtained articles were independently assessed for final inclusion.

### 2.11 Data extraction

Data were independently extracted from studies included in the review. Extracted data included: relevant device, relevant purpose of use, study population, sample size, country, and outcomes relevant to the literature review objectives.

## 3 Results

### 3.1 Review of scientific literature

The search generated 381 references that are possibly relevant to the antimicrobial efficacy and cytotoxicity of superoxidized solutions and their use in wound care. Once titles and abstracts, where available, had been assessed, hard copies of 83 papers were examined, including publications obtained from lists of references. Two systematic reviews (Dumville et al., 2017; Peters et al., 2020) and eight studies were considered relevant for the review of antimicrobial topical treatment of diabetic foot ulcers. Twenty publications were used to extract information on antimicrobial properties of superoxidized solutions and eight to report on biological compatibility.

#### 3.1.1 Superoxidized solutions in the treatment of chronic wounds

The International Working Group on the Diabetic Foot expert panel periodically conducts a systematic review of the published evidence relating to the interventions for managing infection in the diabetic foot (Peters et al., 2012; Peters et al., 2016; Peters et al., 2020). The latest update included 53 studies on the use of surgical procedures, topical antiseptics, negative pressure wound therapy, and hyperbaric oxygen. Of these, three studies discussed the use of superoxidized solutions. In two studies (Martínez-De Jesús, 2007; Piaggesi et al., 2010), using superoxidized water was associated with a better outcome than soap or povidone iodine; however, both studies had a high risk of bias. No benefit has been reported with any other intervention. One additional unblinded study was found comparing superoxidized solution alone and oral levofloxacin with either normal saline or superoxidized solution (Landsman et al., 2011). No significant differences in the rate of clinical success were found. The authors noted that weak trial designs, incomplete reporting, and possible sources of bias limit the generalizability of the evidence. Overall, there is currently no trial data to justify the adoption of any particular therapeutic approach in diabetic patients with infection of either soft tissue or bone of the foot. Dumville et al. (2017), in their Cochrane systematic review, reached a similar conclusion that the relative effects of antimicrobial topical treatments remain uncertain, and no recommendations can be made. Of the 22 randomized controlled trials included in the review, five studies compared superoxidized solutions with either povidone iodine (Piaggesi et al., 2010; Kapur and Marwaha, 2011), normal saline (Hadi et al., 2007; Bowling et al., 2011) or soap (Martínez-De Jesús, 2007). Very low certainty evidence pointed to a slight advantage of the use of antimicrobial topical treatments compared to non-antimicrobial ones (Dumville et al., 2017). Overall, insufficient trial data exist to justify the adoption of any particular therapeutic approach in diabetic foot. The evidence is limited by weak trial designs, incomplete reporting, and possible sources of bias. Additional two single-arm studies with Microdacyn for the treatment of pressure ulcers (Hans, 2022) and diabetic foot ulcers (Walia et al., 2021) were found. These two studies did not affect the overall quality of evidence as presented by Peters et al. (2020), Peters et al. (2016), Peters et al. (2020), and Dumville et al. (2017) (see Table 1).

**TABLE 1:** Superoxidized solutions (SOS) in the treatment of chronic wounds.

Reference	Study design	Population	Interventions	Outcomes	Conclusions
Martínez-De Jesús (2007)	Single-blind RCT	45 patients patients with severe diabetic foot infections	Test group (21): Neutral pH superoxidised aqueous solution (NpHSS)	Fetid odour reduction Infection control Cellulitis reduction Advances from infection to granulating tissue Improvement of skin around the ulcer	Superoxidized water was associated with a better outcome than soap or povidone iodine
			Control group (16): standard care - soap or povidone iodine		
Piaggesi et al. (2010)	RCT	40 patients patients with severe postsurgical lesions of the diabetic foot	Test group A (20): Dermacyn^®^ Wound Care	Ulcer size reduction Amputations Microbiological burden Adverse events Healing rate at 6 months	Superoxidized water was associated with a better outcome than povidone iodine
			Control group B (20): povidon iodine		
Landsman et al. (2011)	Randomized, prospective, multicenter, open-label study	67 patients with diabetic foot ulcers with mild infection	Microcyn Rx Wound Care Saline + Oral levofloxacin Microcyn Rx Wound Care + Oral levofloxacin	Cure Improvement Failure Indeterminate Microbiological response	The differences in clinical success rates were not statistically significant; Microcyn Rx alone had clinical success comparable with saline plus levofloxacin
Kapur and Marwaha (2011)	Retrospective analysis	200 patients with wounds of different origin	Group A (100): superoxidised water (Oxum) Group B (100): povidone iodine (Betadine)	Wound size reduction discharge, pain, odema, redness, granulation tissue, epitheliazation of the wounds	Oxum treated wounds showed reduction in inflammation and their healing earlier than betadine group. Oxum application was safe having no pain and allergic manifestation
Hadi et al. (2007)	Single-center single blinded RCT	100 patients with infected diabetic wounds	Group A: superoxidised water Group B: normal saline	Duration of hospital stay Downgrading of the wound category Wound healing time Need for interventions such as amputation	Statistically significant differences favored superoxidized water with respect to duration of hospital stay, downgrading of the wound category and wound healing time
Bowling et al. (2011)	Prospective, two-center, randomized, controlled, double-blind, pilot study	20 patients with diabetic foot ulcers	Group A: superoxidised water in the Versajet Lavage System Group B: normal saline in the Versajet Lavage System	Reduction of bacterial load Reduction of wound size Adverse events	No significant differences in the reduction of bacterial load and wound size between groups were observed at week 4 of treatment versus baseline
Hans (2022)	Single arm study	50 patients with pressure ulcers	Superoxidized solution and gel (Microdacyn)	PUSH score Healing time Reduction in wound infection Wound size reduction Appearance of granulation tissue and epithelisation Adverse effects	The mean healing time in our study was 5.3 weeks
Walia et al. (2021)	Single arm study	50 patients with diabetic foot ulcers	Superoxidized solution and gel (Microdacyn)	Healing time Duration of hospitalization Wound size reduction Adverse effects	Superoxidized solution was associated with faster healing of ulcers without any major complications

RCT, randomized controlled trial.

#### 3.1.2 Antimicrobial properties of superoxidized solutions

The mechanism of action of superoxidized solutions on bacteria is based on damage to cells by a high oxidation-reduction potential and their lysis by the action of osmotic pressure. Superoxidized solutions contain a mixture of inorganic oxidants such as hypochlorous acid (HClO), hypochlorous acidic ion (ClO-), chlorine (Cl_2_), hydroxide (OH), and ozone (O_3_). Neutral superoxidized solutions contain free oxygen radicals similar to those produced in mitochondria during ATP production and in secretory granules of leukocytes. Superoxidized solutions kill microorganisms directly through their oxidative capacity as they react with the cell wall and membrane and signal protease activation through pH-dependent NADPH oxidase. Exposure of bacteria to oxidative compounds with an ORP between +650 mV and 700 mV induces oxidative stress, resulting in bactericidal effect within a few seconds (Cloete et al., 2009; Armstrong, 2017).

Zinkevich et al. (2000) investigated the mechanism of action of antimicrobial properties of Sterilox, using *E. coli* and analyzing protein and nucleic acid damage. Within 5 min of exposure, the solution destroyed chromosomal and plasmid DNA, RNA, and surface proteins. No intact cells were seen after 5 min of exposure. Within 30 s of exposure, Sterilox entered the cell, causing structural and functional damage to the cell membrane and the cell wall, resulting in swelling. The eventual rupture of the cell wall occurs within 5 min of exposure, causing leakage of cytoplasm and the destruction of proteins, DNA, and RNA (Zinkevich et al., 2000).

HOCl increases oxygenation at wound sites and breaks down biofilm by nonspecifically targeting biomolecules on bacterial cell membranes. HOCL increases permeability of the bacterial cell membrane, damaging the cell integrity. HOCL attacks the microbe cell membrane by dissolving the protective membrane of the biofilm (Gold et al., 2020).

Physiologically, HOCl is produced in the respiratory burst of activated neutrophils. HOCl is a potent oxidant, capable of oxidizing thiol groups and thioethers and halogenating amine groups to form monochloramines and dichloramines. HOCl covalently modifies key amino acid residues of Matrix Metalloproteinase 7 (MMP-7) within the cell. Higher HOCl-to-protein ratios eventually inactivate MMP-7. HOCl exerts a rapid and selective inhibition on RNA and DNA synthesis. It may disrupt membrane/DNA interactions needed for replication, alter the DNA template itself, inactivate enzymes of the replication system, or even inhibit the synthesis of critical proteins required for DNA replication and cell division. HOCl targets methionine residues in proteins of phagocytosed bacteria. The formation of oxidized methionine is strongly associated with bacterial killing (Armstrong et al., 2015).

Superoxidized solutions are effective against a number of aerobic and facultatively aerobic bacteria, anaerobic bacteria, viruses, bacterial spores, bacteriophages and Eukaryotes. They also show good efficacy against biofilms (see Table 2).

**TABLE 2:** Antimicrobial properties of superoxidized solutions reported in literature.

Reference	Products	Microorganisms tested	Summary
Cloete et al. (2009)	Different concentrations of anolyte	*Pseudomonas aeruginosa*, *Staphylococcus aureus*, *Bacillus subtilis* (vegetative) and *Escherichia coli*	The undiluted anolyte was effective in killing all the test bacteria within seconds. When diluted to 10–1, the anolyte killed all the test bacteria except *B. subtilis*. The total elimination of *B. subtilis* by 10^−1^ anolyte dilution occurred within 6 h. Anolyte interfered with the protein composition of E.coli and *P. aeruginosa*, either completely or partially degrading proteins due to oxidative stress
Rossi-Fedele et al. (2010)	Optident Sterilox Electrolyte Solution^®^ irrigation (negative control) Sodium hypochlorite irrigation Sterilox’s Aquatine Alpha Electrolyte^®^ irrigation	*Enterococcus faecalis*	Sterilox’s Aquatine Alpha Electrolyte^®^ showed higher antimicrobial properties compared to the Optident Sterilox Electrolyte Solution^®^ alone. NaOCl was the only solution that consistently eradicated *E. faecalis*
Thorn et al. (2011)	Electrochemically activated solutions	Aerobic/facultative bacteria: *Acinetobacter* spp., Actinobacillus actinomycetemcomitans, *Aeromonas* liquefaciens, *Alcaligenes* faecalis, *Bacillus subtilis*, *Bacillus* cereus, Burkholderia cepacia, *Citrobacter freundii*, *Campylobacter* jejuni, *Escherichia coli*, *Enterobacter* aerogenes, *Enterococcus* spp., VRE, Flavobacter spp., *Haemophilus* influenzae, *Helicobacter pylori*, *Lactobacillus* spp., *Legionella pneumophila*, *Listeria* monocytogenes, *Klebsiella* spp., *Micrococcus* luteus, *Mycobacterium* spp., *Proteus* spp., *Pseudomonas aeruginosa*, *Salmonella* spp., *Serratia marcescens*, *Staphylococcus* spp., MRSA, MRSE, *Streptococcus* spp., Xanthomonas maltophilia Anaerobic bacteria: *Actinomyces* spp., Bifidobacterium bifidum, *Bacteroides fragilis*, Eubacterium lentum, *Fusobacterium* nucleatum, *Peptococcus* niger, *Peptostreptococcus* anaerobius, Prevotella melaninogenica, Porphyromonas spp., Prevotella loeschii, Propionibacterium acnes, Veillonella parvula Bacterial spores: *Bacillus anthracis*, *Bacillus* atrophaeus, *Bacillus* cereus, *Bacillus subtilis*, *Clostridium difficile*, *Clostridium perfringens*, *Streptomyces* spp. Eukaryotes: Aspergillus spp., *Candida* spp., Cryptosporidium parvum oocysts, various environmental fungi	The study lists experimental kill rates determined for electrochemically activated solution anolyte against aerobic, facultative and anaerobic bacteria, bacterial spores, and eukaryotic cells. Kill rates (k) are expressed as log10 colony-forming units (CFU) ml−1 reduction per minute from the viable count and time data points provided within the literature (lowest estimates). Qualitative studies are reported where no quantitative data exist
Ono et al. (2012)	Hypochlorous acid	Standard strains: *P. aeruginosa*, E.coli, Stenotrophomonas maltophilia, A. baumannii, S. typhimuriom, *E. faecalis*, E. faecium, B. subtillis, B. cereus, C. albicans, *A. niger*, Phage Q β Clinical isolates: P.aeruginosa, A. baumannii, *S. aureus* (MRSA and MSSA), *E. faecalis*, E. faecium, E. avium, C. albicans, C. grabrata, C. krusei, *C. tropicalis*	The weak acid hypochlorous solution had an excellent microbicidal effect against a broad microbicidal spectrum of standard strains and clinical isolates in a short time. The microbicidal effects of hypochlorous solutions did not depend on the available chlorine concentration but on the HClO concentration
Mena-Mendivil et al. (2013)	Microdacyn 60, OxOral, sodium hypochlorite 5.25%	*Streptococcus* sobrinus, Porphyromona gingivalis, *Streptococcus* intermedius, Tanerella forsytensis, *Enterococcus faecalis*	Sodium hypochlorite (NaOCl) is commonly used solution for root canal treatment. NaOCl is toxic to periradicular tissues and can cause necrosis of support tissues. This study compared the antimicrobial effect of Microdacyn 60^®^, OxOral^®^, and NaOCl 5.25% against typical anaerobic pathogens present in the root canal. Thirty-three extracted teeth were inoculated with a mixture of bacteria and incubated for 7 days. After irrigation with the test solutions, samples were taken and placed in an Eppendorf tube for incubation. Samples were taken for a bacterial identification and count after 7 days. NaOCl and OxOral eliminated all bacteria. In the Microdacyn 60 group, *E. faecalis* showed the highest resistance. NaOCl 5.25% had a greater antibacterial effect against anaerobes typically present in the root canal
Torres-Capetillo et al. (2013)	Neutral super-oxidized electrolyzed antimicrobial gel (EsteripHarma Mexico, SA de CV, Mexico City, Mexico) and chlorhexidine digluconate 0.12% (Farmacia Morlan, Toledo, Spain)	*Streptococcus* intermedius, Porphyromonas gingivalis	This study compared the antimicrobial efficacy of neutral super-oxidized electrolyzed gel and chlorhexidine digluconate against *Streptococcus* intermedius and Porphyromonas gingivalis. Thirty sterile orthodontic mini-implants were impregnated with test products for 10 min, then immersed in bacterial culture and incubated for 24 h. Samples were taken to count colony-forming units (CFU), and to determine bacterial absorbance and concentration as well as cytotoxicity. Superoxidized gel had a lower cytotoxicity and lower inhibitory effect on both *S. intermedius* and P. gingivalis compared to chlorhexidine. While super-oxidized gel had inhibitory effect on bacterial growth around the mini-implant, chlorhexidine digluconate was bactericidal
Gunaydin et al. (2014)	Medilox^®^ super-oxidized water	Standard strains: *Acinetobacter* baumannii 19606, *Escherichia coli* 25922, *Enterococcus faecalis* 29212, *Klebsiella pneumoniae* 254988, *Pseudomonas aeruginosa* 27853, *Staphylococcus aureus* 29213 Clinical isolates: *Acinetobacter* baumannii, *Escherichia coli*, vancomycin-resistant *Enterococcus* faecium, *Klebsiella pneumoniae*, *Pseudomonas aeruginosa*, methicillin-resistant *Staphylococcus aureus*, *Bacillus subtilis*, Myroides spp. Yeasts: *Candida* albicans, *Candida tropicalis*, *Candida* parapsilosis, *Candida* glabrata, *Candida* krusei, *Candida* lusitaniae, Trichosporon spp. Molds: Aspergillus fumigatus, Aspergillus flavus, Aspergillus niger	This study investigated the *in-vitro* antimicrobial activity of different concentrations of Medilox^®^ [Soosan E and C, Korea] super-oxidized water against a variety of standard strains and clinical isolates. Antimicrobial activities of different concentrations (1/1 to 1/100) were measured at different exposure times (1–30 min). Medilox^®^ was effective against all standard strains, all clinical isolates, and all yeasts at 1/1 dilution in more than 1 min and against Aspergillus flavus at 1/1 dilution in more than 2 min. Certain molds needed 5 min of exposure
Sakarya et al. (2014)	stabilized HOCl solution for all standard microorganisms was 1/64 dilution and for clinical isolates it ranged from 1/32 to 1/64 dilutions	Standard strains: *S. aureus* ATCC35556, *P. aeruginosa* ATCC 15692, C. albicans (ATCC 90028) Clinical isolates: *S. aureus*, *P. aeruginosa*, C. albicans	Topical antiseptics in chronic wounds remain are successful in microbial eradication, but their cytotoxcity may hinder wound healing. HOCl has good antimicrobial properties and favorable effect on the migration of keratinocytes and fibroiblasts. This study investigated the effect of stabilized hypochlorous acid (HOCl) on killing rate, biofilm formation, antimicrobial activity within biofilm against standard strains and clinical isolates of *S. aureus*, *P. aeruginosa*, and C. albicans, and the effect on fibroblasts and keratinocytes. The minimal bactericidal concentration (MBC) of HOCl solution was 1/64 against all standard strains. The MBC against clinical isolates ranged from 1/32 to 1/64 dilutions. All microorganisms were killed within seconds. The effective dose for biofilm impairment ranged from 1/32 to 1/16 for for standard strains and clinical isolates
Armstrong et al., 2015 (WHO Application)	Neutral electrolytically activated water solutions (NEW)	Aerobic/facultative bacteria: *Acinetobacter* spp., Actinobacillus actinomycetemcomitans, *Aeromonas* liquefaciens, *Alcaligenes* faecalis, *Bacillus subtilis*, *Bacillus* cereus, Burkholderia cepacia, *Citrobacter freundii*, *Campylobacter* jejuni, *Escherichia coli*, *Enterobacter* aerogenes, *Enterococcus* spp., VRE, Flavobacter spp., *Haemophilus* influenzae, *Helicobacter pylori*, *Lactobacillus* spp, *Legionella pneumophila*, *Listeria* monocytogenes, *Klebsiella* spp., *Micrococcus* luteus, *Mycobacterium* spp., *Proteus* spp., *Pseudomonas aeruginosa*, *Salmonella* spp., *Serratia marcescens*, *Staphylococcus* spp., MRSA, MRSE, Stentotrophomonas maltophilia, *Streptococcus* spp., Xanthomonas maltophilia Anaerobic bacteria: *Actinomyces* spp., Bifidobacterium bifidum, *Bacteroides fragilis*, *Clostridium difficile*, Eubacterium lentum, *Fusobacterium* nucleatum, *Peptococcus* niger, *Peptostreptococcus* anaerobius, Prevotella melaninogenica, Porphyromonas spp., Prevotella loeschii, Propionibacterium acnes, Veillonella parvula Viruses: FCV 2280, Flu A H1N1, Flu A H5N1, Flu A H9N2, Flu A H3N1, HIV 1, HSV 1, HSV 2, Norovirus, Polio 1, Rhino A1, RSV, WNV Bacterial Spores: *Bacillus anthracis*, *Bacillus* atrophaeus, *Bacillus* cereus, *Bacillus subtilis*, *Clostridium difficile*, *Clostridium perfringens*, *Streptomyces* spp. Bacterophages: Bacteriophage Qβ Eukaryotes: Aspergillus spp., *Candida* spp., Cryptosporidium parvum oocysts, various environmental fungi Biofilms 24h: *Staphylococcus aureus*, *Pseudomonas aeruginosa*, *Candida* albicans	NEW has a broad biocidal effect against bacteria, viruses, fungi, spores, eukaryotes, and biofilms Following the disruption of the cellular membrane, the low osmolarity of NEW, typically around 13 mOsmol/L, causes cell death by osmotic rupture. Since the antimicrobial efficacy of NEW is essentially rapid osmotic shock, it is not believed to be susceptible to the development of antimicrobial resistance because of its extremely rapid physical mode of action and not cytotoxic mode of action
Armstrong et al., 2015 (EO)	Hypochlorous acid	Standard strains: E.coli NCTC 9001, E.coli NCTC 12900, Aspergillus niger 16404, *Candida* albicans 10231, 90028, Corznebacterium amycolatum 49368, E. aerogenes 51697, E.coli 25922, Haemophillus influenzae 49144, *Klebsiella pneumoniae* 10031, *Micrococcus* luteus 7468, *Proteus mirabilis* 14153, *Pseudomonas aeruginosa* 15692, 27853, *Serratia marcescens* 14756, *S. aureus* 29213, 35556, S. epidermidis 12228, S. haemolyticus 29970, S. hominis 27844, S saprophyticus 35552, S pyogenes 49399, MRSA 33591, VREF 51559 Clinical isolates: E.coli 0157, MRSA, *Candida* albicans, *Bacillus subtilis* spores, *Enterococcus faecalis*, *Pseudomonas aeruginosa*, *S. aureus*	The antimicrobial activity of HOCl is comparable to other antiseptics. *In-vitro* studies show good efficacy against a number of standard strains and clinical isolates. Significant advantage of HOCl is the absence of cytotoxicity
Gray et al. (2016)	Hypochlorous acid	MRSA	The decolonization from MRSA is typically performed by baths with mupirocin and chlorhexidine. This regimen is not feasible for burn patients since chlorhexidine shall not be used on breached skin and mucosa. Gray et al. (2016) investigated the efficacy of batch containing muciprocin combined with hypochlorous acid for decolonization of hospital acquired MRSA in a burn intensive care unit. The study showed significant decrease in MRSA infections in burn patients
Al-Mualla et al. (2018)	Dermacyn^®^ Wound Care Solution and Microcyn^®^ Hydrogel (both Oculus Innovative Sciences)	*Staphylococcus aureus* MRSA, *Enterococcus faecalis* VRE, *Staphylococcus aureus*, *Escherichia coli*, *Acinetobacter* baumannii, *Bacteroides fragilis*, *Candida* albicans, *Enterobacter* aerogenes, *Enterococcus* faecium VRE - MDR, Haemophilius influenzae, *Klebsiella* oxytoca MDR, *Klebsiella pneumoniae*, *Micrococcus* luteus, *Proteus mirabilis*, *Pseudomonas aeruginosa*, *Serratia marcescens*, *Staphylococcus* epidermidis, *Staphylococcus* haemolyticus, *Staphylococcus* homins, *Staphylococcus* saprophyticus, *Streptococcus* pyogenes	Super-oxidized solutions have a broad spectrum antimicrobial effect (bactericidal, virucidal, fungicidal, and sporicidal), which helps reduce the wound microbial burden and aids in biofilm removal
Cai et al. (2018)	Chlorite-based disinfectants, including sodium hypochlorite (SH), chlorine dioxide (CD), strongly acidic electrolyzed water (StAEW), and neutral electrolyzed water (NEW)	Biofilm: *Enterobacter cloacae*, *Klebsiella* oxytoca, and *Citrobacter freundii*	Bacterial biofilms on equipment are a common source of cross-contamination. This study investigated the effect of sodium hypochlorite (NaOCl), chlorinedioxide, strongly acidic electrolyzed water, and neutral electrolyzed water on biofilms formed by *E. cloacae*, K. oxytoca, and *Citrobacter freundii*. *E. cloacae* biofilms were the most resistant to disinfectants. NaOCl was the most effective disinfectant in disrupting *E. cloacae* biofilm
Harriott et al. (2019)	Vashe and PhaseOne	Bacterial biofilms: Multiple standard strains of MSSA, MRSA, *E. faecalis*, S. pyogenes, E.coli, *K. pneumoniae*, *P. aeruginosa*, *E. cloacae*, *P. mirabilis*, S. maltophilia Fungal biofilms: C. albicans, C. glabrata, C. parapsilosis	Superoxidized solutions Vashe and PhaseOne have excellent bactericidal and fungicidal properties. Sulfamylon had minimal activity against biofilm Vashe and PhaseOne eliminated most biofilms within 1 or 10 min. No current consensus exists for the treatment of biofilm affecting chronic wounds or medical devices. The results of this study suggest that hypochlorous acid–based wound solutions are superior to mafenide in eliminating biofilm
Schwarzer et al. (2019)	Hypochlorous acid	Biofilm	Topical agents have been widely adopted in clinical practice to manage biofilm in chronic wounds, despite limited evidence *in vivo* to support their effectiveness. This study evaluated the evidence for topical agents used in chronic wounds with biofilm. The systematic review included 43 articles. *In vitro* testing accounted for 90% of evidence (39 studies). Five animal studies (of which one involved hypochlorous acid) and three human *in vivo* studies were also included. The studies included 44 different topical agents, most commonly silver, iodine and polyhexamethylene biguanide (PHMB). There is insufficient evidence from human studies to recommend any of the topical agents over others
Herruzo and Herruzo (2020)	Chlortech, Vetericyn VF-skin care, Microdacyn60, Betadine, Cristalmina, Perioaid, Lacer-chlorhexidine, Octenisept-Farblos, Prontosan	E. faecium; S. epidermidis, *S. aureus*; *Morganella morganii*; *Enterobacter cloacae*, *P. aeruginosa*, *Candida* albicans, Torulopsis glabrata	The study aimed to compare the antimicrobial efficacy of 13 antiseptics including ClHO (Clortech R) with hypochlorous acid, chlorhexidine and povidon iodine on 8 microorganisms on organic germ carriers. 1% Chlorhexidine had the highest microbicidal effect at 1 min. ClHO (300 or 500 mg/L) is a good antiseptic tsuitable for the use on wounds and mucous membranes for 5–10 min. ClHO (1,500 mg/L) remains effective against biofilm
Aranke et al. (2021)	Super-oxidized water	HIV, Myobacterium tuberculosis, *Candida* albicans, and *Pseudomonas aeruginosa*, SARS-CoV-2	Super-oxidized water is used medically as a disinfectant for simple surfaces, root canals, wounds, and reusable medical devices. In minutes, super-oxidized water is proposed to be effective against the human immunodeficiency virus, *Mycobacterium tuberculosis*, *Candida* albicans, and *Pseudomonas aeruginosa*
Jimenez-Gonzalez et al. (2021)	Calcium Hydroxide Combined with Electrolyzed Superoxidized Solution at Neutral pH (OxOral^®^)	*Enterococcus faecalis*	The study evaluated the effect of the combination of calcium hydroxide and a neutral superoxidized solution (OxOral^®^) on *Enterococcus faecalis*. Sixty human teeth were used. The root canals were infected and randomized into treatment with normal saline, normal saline plus calcium hydroxide, OxOral^®^, and OxOral^®^ combined with calcium hydroxide. OxOral^®^ plus calcium hydroxide permanently reduced bacterial growth at days 1, 6, 12, and 18, retaining alkaline pH
Savadkouhi et al. (2021)	Super-oxidized water	Biofilm: *Enterococcus faecalis*	The study compared the effect of superoxidized water and sodium hypochlorite on the elimination of *E. faecalis* biofilm from the root canal The solutions were tested on 32 extracted human incisors. The specimens were sterilized and inoculated with bacterial suspension. The teeth were randomized into four groups: positive control (irrigation with normal saline), negative control (tooth without biofilm), intervention 1 (sodium hypochlorite) and intervention 2 (superoxidized water). Based on this study, the sodium hypochlorite reduced biofilm thickness and CFU/mL by 100%. Superoxidized water reduced biofilm thickness by 98% and CFU/mL by 90%
Salisbury and Percival (2019)	Polihexanide (PHMB), Octenidine HCI based wound irrigation solution and electrolysed water based wound care solution	Biofilm: *Staphylococcus aureus*, *Pseudomonas aeruginosa* and a multispecies biofilm	Electrolysed water is commonly used in clinical practice to control bioburden in wounds. The evidence on the efficacy of electrolyzed irrigation solutions against biofilm is limited. This study assessed the efficacy of electrolysed water on *S. aureus* and *P. aeruginosa* biofilms *in vitro*. Electrolysed water reduced biofilm in all models following a 15 min contact time. Based on cytotoxicity tests on fibroblasts, a 50% and 25% dilution of the electrolysed water formulation was non-cytotoxic. New electrolysed water product effectively removed biofilm after a short exposure time, making it an attractive option for chronic, non-healing wounds

#### 3.1.3 Biocompatibility of superoxidized solutions

Wound irrigation solutions offer the first line of defense against microbial colonization of the wound. Intimate contact with viable wound cells is inevitable, so it is vital that wound irrigation solutions demonstrate good cell compatibility. Cytotoxic effects of a wound dressing would reduce the viability, proliferation, and migration of cells involved in the wound healing process, leading to decreased healing rate. Cytotoxicity data derived from *in vitro* studies must be interpreted with caution, as any cytotoxic effects observed in cultured cell types can be magnified and may not reflect the clinical setting. Overall, the evidence points to minimal or low cytotoxicity of superoxidized solutions. Superoxidized solutions do not induce skin sensitization or irritation in animal studies despite the high oxido-reduction potential (ORP) and antimicrobial activity (see Table 3).

**TABLE 3:** Overview of cytotoxicity of superoxidized solutions identified in literature.

Author, year	Product	Tests	Result
Landa-Solis et al. (2005)	Microcyn	Direct cytotoxic effect on MT-2 cells diluted serially (10^−1^ to 10^−5^)	No cytopathic effect
Gutiérrez (2006)	Microcyn	cytotoxicity test on fibroblasts was executed in accordance with ISO 10993–5:1999	No cytotoxicity No genotoxicity No accelerated aging
Gonzalez-Espinosa et al. (2007)	Microcyn	cytotoxicity test on fibroblasts as measured by 8-hydroxy-2#deoxyguanosine (8-OHdG) adducts, nucleic acid stability and ageing process	Microcyn is significantly less cytotoxic than antiseptic hydrogen peroxide concentrations (i.e. 880 mM) and that, *in vitro*, it does not induce genotoxicity or accelerated ageing
le Duc et al. (2007)	Dermacyn	Two different human skin substitutes (HSSs) Detrimental changes in histology, metabolic activity (MTT assay) and RNA staining of tissue sections	Not cytotoxic for either HSS or autograft. MTT levels were >70% (unexposed cultures = 100%), which implies a very mild cytotoxic effect of these antiseptics on all three models
Gomi et al. (2010)	Hypochlorous acid	Mitogenic assay (MTT) and alkaline phosphatase (ALPase) activity in pulp cells	Hypochlorous acid damaged the pulp cells. The cellular disorder was not found in the 10- or 1.000-times dilution
Ortega-Pena et al. (2017)	Microdacyn^®^ (Morepharma, Mexico) Vashe^®^ (SteadMed Medical, TX, United States)	Human fibroblast cytotoxicity Mitogenic assay (MTT)	Chlorine-releasing agents exhibited immediate anti-biofilm effects in the short term, with lesser cytotoxicity than agents prepared from more stable compounds, such as biguanide or modified diallyl disulfide-oxide, which, conversely, have better long-term effectiveness
Salisbury and Percival (2019)	electrolysed water produced on site	Indirect cytotoxicity tests in accordance with ISO 10993–5 ASTM 895–11 Standard Test Method for Agar Diffusion Cell Culture Screen for Cytotoxicity	Electrolyzed water (EW) EW 100%: zone index 3 (Cytotoxic) Lysis index: 3 (Cytotoxic) EW 75%: zone index 3 (Cytotoxic) Lysis index: 1 (Non-cytotoxic) EW 50%: zone index 2 (Non-cytotoxic) Lysis index: 1 (Non-cytotoxic) EW 25%: zone index 2 (Non-cytotoxic) Lysis index: 1 (Non-cytotoxic)
Salisbury and Percival (2019)	Microdacyn^®^ (Bamboo Healthcare GmbH, Germany) Granudacyn^®^ (SastoMed GmbH, Germany) Veriforte™ Mediset Clinical Products GmbH, Germany	human keratinocytes human skin fibroblasts XTT assay	Veriforte™, Microdacyn^®^ and Granudacyn^®^ demonstrated no cytotoxicity for human keratinocytes (HaCaT) and skin fibroblasts (BJ) within 15 min of exposure

### 3.2 Survey respondents

The Manufacturer collected 237 survey forms from 81 different healthcare facilities, nursing homes, and outpatient clinics located in 57 towns and cities around the Czech Republic. The majority of forms (214) were filled in and signed by nurse practitioners.

### 3.3 Characteristics of the treated population

**FIGURE 1:**
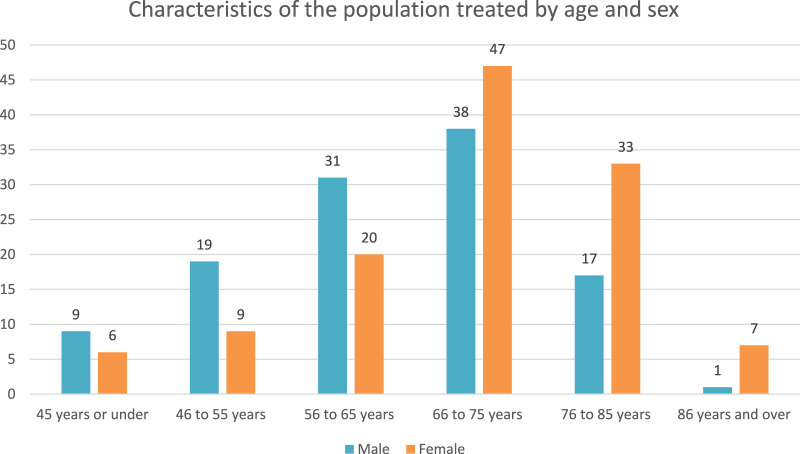
Patient characteristics by age and sex.

Of the 237 patients, 115 were male and 122 were female. More men were represented in the younger categories than women (see Figure 1).

**FIGURE 2:**
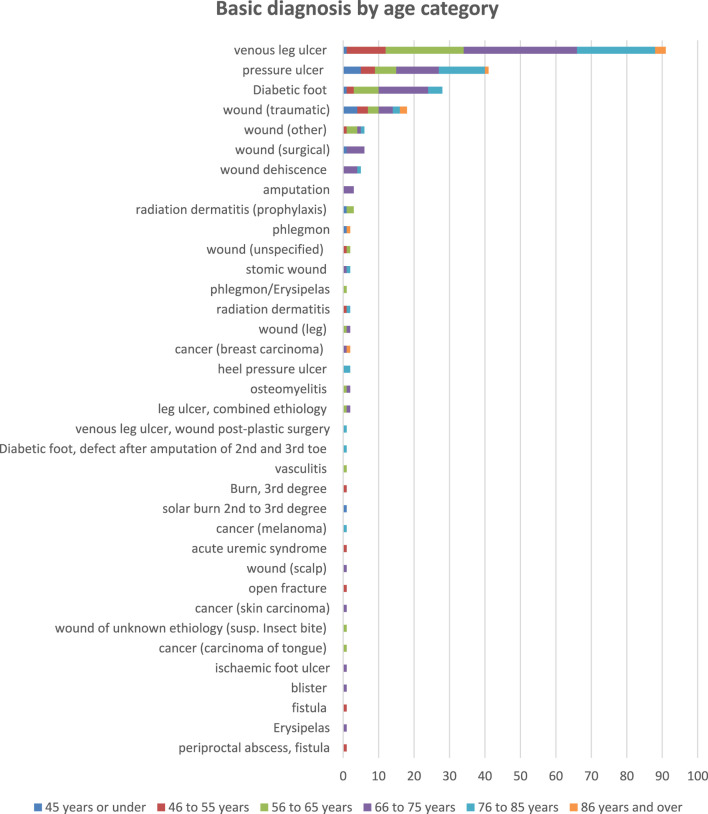
Patient characteristics: Basic diagnosis by age category.

The most common basic diagnoses were venous leg ulcer (91; 38%), pressure ulcer (41; 17%), diabetic foot ulcer (28; 12%), and traumatic wound (18; 8%) (see Figure 2).

**FIGURE 3:**
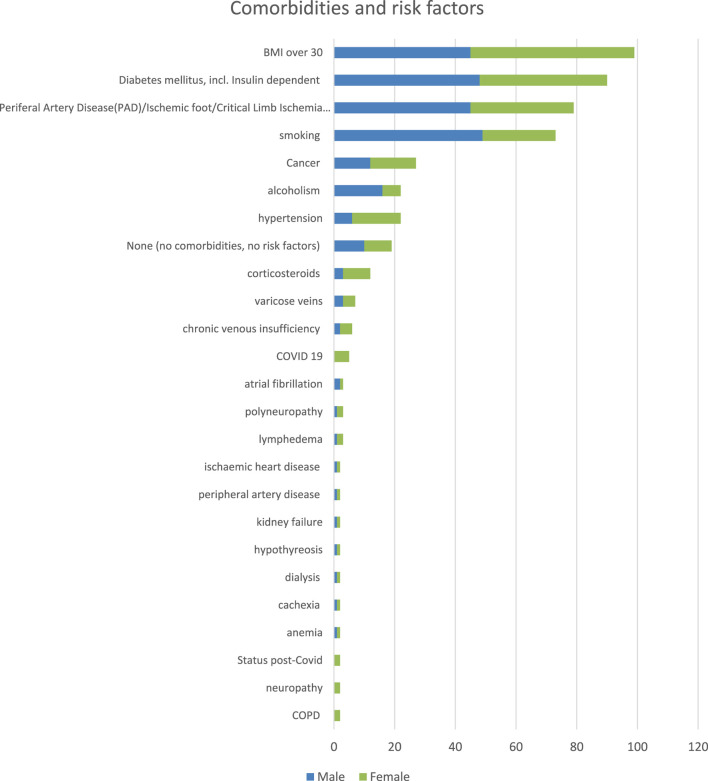
Patient characteristics: Comorbidities and risk factors.

Of the 237 patients, 99 (42%) had body mass index (BMI) over 30, 90 (38%) suffered from diabetes mellitus, 79 (33%) had peripheral artery disease (PAD), to include ischemic foot or critical limb ischemia (CLI), 73 (31%) smoked tobacco, 27 (11%) had cancer, 22 (9%) were alcoholics, 22 (9%) had hypertension, 12 (5%) were on corticosteroid treatment, 7 (3%) had varicose veins, 6 (3%) suffered from chronic venous insufficiency, and 5 (2%) had COVID-19. Only 19 (8%) of the 237 included patients had no reported comorbidities and no risk factors (see Figure 3).

### 3.4 Time lag between the appearance of the wound and initial examination

The time lag between the injury and the first visit when treatment with DebriEcaSan Alfa was initiated varied greatly between the patients. Only 39 (16%) patients presented with their wounds within a week. Another 94 (40%) patients came with wounds older than 1 week but within 3 months (See Table 4). A significant number of patients (55, 23%) presented with chronic, non-healing wounds that were older than 3 months. Of these, 27 patients had wounds older than 2 years at initial examination, including 5 patients whose wounds lasted 10 years or more. There is no difference between men and women when it comes to the time lag between the first appearance of the wound and the time of initial examination. The wounds that were older than 3 months at the time of presentation (55 patients) tended to be complex, large, and with symptoms of infection (see Table 5). The 49 patients who presented with wounds where the time lag was unknown had significant comorbidities and risk factors and wounds that were often large, deep, and with symptoms of infection (see Table 6).

**TABLE 4:** The time lag between the injury and the first visit.

	Male	%	Female	%	Total	%
same/following day	6	5%	6	5%	12	5%
3 days or less	4	3%	4	3%	8	3%
4 days to 1 week	11	9%	8	7%	19	8%
1–2 weeks	11	9%	11	9%	22	9%
less than a month (2 weeks–1 month)	11	9%	9	7%	20	8%
less than 3 months (1–3 months)	25	21%	27	22%	52	22%
less than 1 year (3 months–1 year)	10	9%	13	11%	23	10%
1–2 years	2	2%	2	2%	4	2%
2 or more years	15	13%	13	11%	28	12%
Not available	20	17%	29	24%	49	21%
Total	115	100%	122	100%	237	100%

**TABLE 5:** Characteristics of wounds older than 3 months at first presentation.

Patient	Time lag	Basic diagnosis	Comorbidities	Risk factors	Affected structures	Symptoms of infection	Microbiology	Wound area cm^2^	Wound depth cm	Wound volume cm^3^
48 M	>10 years	Venous leg ulcer	Incontinence, psychiatric diagnosis	None	Muscle	Malodor after removal of dressing, occasional pain, serous discharge, undermined wound bed	None	55	>1	28
51M	>10 years	Venous leg ulcer	PAD	Smoking	Subcutaneous tissue	Malodor after removal of dressing, occasional pain, serous discharge, biofilm	G- (Unspecified)	150	1–2	150
52 F	394 days	Diabetic foot	Diabetes	BMI >30, Smoking	Subcutaneous tissue, Muscle	serous discharge, biofilm	None	25	2–3.9	55
52 M	>10 years	Venous leg ulcer	None	None	Subcutaneous tissue	Malodor after removal of dressing, occasional pain, serous discharge, biofilm	G- (Unspecified)	300	1–2	450
54 M	345 days	Venous leg ulcer	varicose veins	BMI >30	Subcutaneous tissue	Intense malodor, continuous pain, purulent discharge, undermined wound bed	G- (Unspecified)	36	>1	14
55 F	6 months	Fistula	None	BMI >30	Subcutaneous tissue, Muscle	Malodor after removal of dressing, purulent discharge	MRSA	1	>6	10
56 M	5 years	Venous leg ulcer	PAD	None	Subcutaneous tissue, Muscle	Malodor after removal of dressing, occasional pain, serous discharge, biofilm	None	53	>1	26
57 M	116 days	Pressure ulcer	status post serious car accident	None	Muscle	Malodor after removal of dressing, pain during dressing change, purulent discharge (xx), undermined wound bed	G+ (Unspecified)	24	2–3.9	72
57 M	6 months	Wound (traumatic)	PAD	Smoking, alcoholism		pain during dressing change, serous discharge	None	3	>1	1
58 F	156 days	Venous leg ulcer	PAD	None	Subcutaneous tissue	Occasional pain, serous discharge, biofilm	None	32	1–2	48
58 M	Several years (2 or more)	Cancer (carcinoma of tongue)	Cancer	Smoking			None	10	0	0
60 F	2 years	Wound (unspecified)	None	BMI >30, corticosteroids	Subcutaneous tissue, Muscle	Malodor through dressing, continuous pain, purulent discharge, undermined wound bed	*Proteus mirabilis*, Strept. Beta-hemolytic group C, *Morganella*, *P. aeruginosa*	n/a	1–2	not stated
62 F	121 days	Venous leg ulcer	varicose veins, leg edema	BMI >30	Subcutaneous tissue	Malodor after removal of dressing, pain during dressing change, serous discharge (xxx), biofilm	None	240	>1	120
62 M	>3 years	Venous leg ulcer	Diabetes, PAD	BMI >30	Subcutaneous tissue	Malodor through dressing, continuous pain, purulent discharge (xx), undermined wound bed	None	80	>1	24
62 F	12 years	Venous leg ulcer	None	BMI >30	Subcutaneous tissue, Muscle	None, pain during dressing change, serous discharge (xx), biofilm	*P. aeruginosa*	2	>1	1
62 M	4 months	Venous leg ulcer	PAD	Smoking	Subcutaneous tissue	Malodor after removal of dressing, occasional pain, serous discharge (xx)	G+ (Unspecified)	48	>1	19
63 M	295 days	Venous leg ulcer	Diabetes, PAD	Smoking	Subcutaneous tissue, Muscle	Intense malodor, occasional pain, bloody discharge, necrosis/gangrene	MRSA	400	2–3.9	1,000
63 F	304 days	Vasculitis	Diabetes, hypothyreosis, hypertension, chronic pulmonary obstruction disease	BMI >30, corticosteroids	Subcutaneous tissue, Muscle, tendon	Intense malodor, continuous pain, purulent discharge, necrosis/gangrene	None	52	1–2	52
63 M	368 days	Venous leg ulcer	varicose veins	Smoking	Subcutaneous tissue	Malodor after removal of dressing, occasional pain, purulent discharge, undermined wound bed	None	20	>1	4
63 M	2 years	Venous leg ulcer	PAD	BMI >30, alcoholism	Subcutaneous tissue, Muscle	Malodor after removal of dressing, occasional pain, serous discharge, biofilm	Other (Unspecified)	84	>1	25
64 M	>10 years	Venous leg ulcer	Diabetes	BMI >30	Subcutaneous tissue	Malodor after removal of dressing, pain during dressing change, serous discharge, undermined wound bed	None	300	2–3.9	600
65 M	More than 3 months	Venous leg ulcer	PAD	BMI >30, Smoking	Subcutaneous tissue	Malodor through dressing, continuous pain, serous discharge, biofilm	MRSA	80	1–2	120
65 F	Several months	Venous leg ulcer	PAD, celiac disease, malnutrition	corticosteroids	tendon	Malodor after removal of dressing, continuous pain, purulent discharge, undermined wound bed	None	50	0	0
66 F	2 years	Venous leg ulcer	varicose veins, lymphedema	BMI >30	Subcutaneous tissue	Intense malodor, continuous pain, purulent discharge, undermined wound bed	G- (Unspecified)	50	>1	20
66 M	More than 5 years	Venous leg ulcer	PAD	None	Subcutaneous tissue	Malodor after removal of dressing, continuous pain, purulent discharge, undermined wound bed	G-/G+ (Unspecified)	100	>1	50
67 F	132 days	Venous leg ulcer	PAD, COPD	BMI >30, Smoking	Subcutaneous tissue, Muscle	Malodor after removal of dressing, continuous pain, purulent discharge, necrosis/gangrene	None	150	>1	30
67 M	More than 5 years	Venous leg ulcer	Diabetes, PAD	BMI >30	Subcutaneous tissue	Intense malodor, occasional malodor, purulent discharge, undermined wound bed	None	180	not stated	not stated
68 F	233 days	Pressure ulcer	Cancer	corticosteroids	Subcutaneous tissue, Muscle	Intense malodor, occasional malodor, bloody discharge, necrosis/gangrene	None	15	1–2	27
68 M	3 years	Venous leg ulcer	PAD	BMI >30	Subcutaneous tissue	Malodor after removal of dressing, occasional malodor, serous discharge, biofilm	None	48	>1	14
68 M	Several months	Venous leg ulcer	ischaemic heart disease	BMI >30, Smoking	Subcutaneous tissue	pain during dressing change, serous discharge (x), biofilm	None	15	>1	8
69 F	100 days	Cancer (breast carcinoma)	Cancer	None	Muscle	Intense malodor, purulent discharge, undermined wound bed	None	48	4–5.9	240
69 M	406 days	Blister	PAD	BMI >30		serous discharge	None	6	not stated	not stated
69 F	2 years	Cancer (skin carcinoma)	Cancer	None	Subcutaneous tissue	Malodor after removal of dressing, occasional malodor, serous discharge, biofilm	E.coli, *S. aureus*	3	>1	1
71 F	More than 5 years	Venous leg ulcer	Diabetes	None		occasional malodor, biofilm	None	35	not stated	not stated
71 F	Several years	Venous leg ulcer	Diabetes, Cancer	None	Subcutaneous tissue	Malodor through dressing, occasional malodor, purulent discharge, undermined wound bed	G+ (Unspecified)	190	1–2	190
72 M	122 days	Wound (leg)	None	None	Subcutaneous tissue	Malodor after removal of dressing, pain during dressing change, serous discharge (xx), biofilm	None	2	>1	1
72 M	>5 years	Venous leg ulcer	Diabetes	BMI >30, Smoking	Muscle	Intense malodor, occasional malodor, purulent discharge (xx), undermined wound bed	G- (Unspecified)	120	1–2	216
73 F	>5 years	Venous leg ulcer	Diabetes	None	Subcutaneous tissue	Malodor after removal of dressing, pain during dressing change, bloody discharge (xxx), undermined wound bed	None	200	>1	100
74 M	136 days	Venous leg ulcer	None	Smoking	Subcutaneous tissue	Intense malodor, occasional malodor, purulent discharge	None	700	2–3.9	1,400
74 M	233 days	Venous leg ulcer	None	BMI >30	Subcutaneous tissue	pain during dressing change, serous discharge, biofilm	None	23	>1	5
75 F	762 days	Wound (scalp)	None	None		undermined wound bed	None	25	>1	3
76 F	Several years	Venous leg ulcer	PAD	BMI >30	Subcutaneous tissue, Muscle	Malodor through dressing, continuous pain, purulent discharge, necrosis/gangrene	*Alcaligenes* faecalis	50	>1	20
77 F	400 days	Venous leg ulcer	PAD	Smoking, corticosteroids	Muscle	Malodor through dressing, continuous pain, purulent discharge, undermined wound bed	None	24	>1	10
78 M	2.5 years	Venous leg ulcer	Diabetes, PAD	BMI >30	Subcutaneous tissue	occasional malodor, serous discharge, biofilm	None	6	>1	1
78 M	2 years	Venous leg ulcer	Varicose veins, lymphedema	BMI >30	Skin	Malodor intense malodor, continuous pain, purulent discharge, undermined wound bed	G-/G+ (Unspecified)	25	>1	8
78 M	2 years	Venous leg ulcer	Diabetes, PAD, Cancer, respiratory failure, covid pneumonia	BMI >30	Subcutaneous tissue	Pain during dressing change, purulent discharge, biofilm	None	28	>1	Not stated
81 F	Several months	Venous leg ulcer	None	BMI >30	Subcutaneous tissue, Muscle	Malodor after removal of dressing, occasional malodor, serous discharge, biofilm	MLSB	234	>1	94
82 F	>3 years	Venous leg ulcer	None	Smoking	Subcutaneous tissue	Malodor through dressing, occasional malodor, serous discharge, undermined wound bed	None	182	>1	91
82 F	2 years	Venous leg ulcer	None	BMI >30	Subcutaneous tissue	Malodor through dressing, continuous pain, purulent discharge, necrosis/gangrene	*P. aeruginosa*	55	>1	11
83 F	121 days	Venous leg ulcer	PAD, venous insufficiency	BMI >30	Subcutaneous tissue	Intense malodor, continuous pain, serous discharge, undermined wound bed	None	120	1–2	120
84 F	120 days	Venous leg ulcer	Diabetes	Corticosteroids	Muscle	Intense malodor, continuous pain, serous discharge, undermined wound bed	None	24	4–5.9	96
84 F	126 days	Diabetic foot	Diabetes	BMI >30	Muscle	Malodor after removal of dressing, continuous pain, serous discharge, undermined wound bed	None	6	2–3.9	14
85 F	Several years	Venous leg ulcer, wound post-plastic surgery	Varicose veins	None	Subcutaneous tissue	Pain during dressing change, serous discharge	None	104	0	0
92 F	Several months	Venous leg ulcer	Diabetes	None	Subcutaneous tissue	Pain during dressing change, serous discharge, undermined wound bed	None	48	>1	38
94 F	1 year	Cancer (breast carcinoma)	None	None	Subcutaneous tissue		None	1	1–2	1

PAD, peripheral artery disease; BMI, body mass index; COPD, chronic obstructive pulmonary disease; MRSA, Methicillin-resistant *Staphylococcus aureus*.

**TABLE 6:** Characteristics of wounds where time lag between wound first appearance and initial presentation was not known.

Patient	Time lag	Basic diagnosis	Comorbidities	Risk factors	Affected structures	Symptoms of infection	Microbiology	Wound area cm^2^	Wound depth cm	Wound volume cm^3^
60 F	N/A	Radiation dermatitis (prophylaxis)	Cancer	None	Skin	None	None	4,900	0	0
50 M	N/A	Pressure ulcer	None	Smoking, alcoholism	Skin, subcutaneous tissue, muscle, tendon, bone	Necrosis/gangrene, purulent discharge, intense malodor	None	30	2–3.9	90
59 F	N/A	Radiation dermatitis (prophylaxis)	Cancer	None	Skin	None	None	1,600	0	0
84 M	N/A	Venous leg ulcer	Prostate cancer, atrial fibrilation, hypertension	None	Skin subcutaneous tissue	Necrosis/gangrene	*S. aureus*, *Klebsiella pneumoniae*	38.5	>1	19
45 F	N/A	Radiation dermatitis (prophylaxis)	Cancer	None	Skin	None	None	900	0	0
69 F	N/A	Leg ulcer, combined ethiology	Polymorbid, cachetic	BMI >30	Skin subcutaneous tissue, muscle, tendon joint bone	Undermined wound bed, purulent discharge, continuous pain, malodor upon removal of dressing	None	900	Not stated	Not stated
55 F	N/A	Radiation dermatitis	Cancer	BMI >30, smoking	Skin	Occasional pain, intense malodor	None	100	0	0
45 F	N/A	Pressure ulcer	Coma	Alcoholism	Skin, subcutaneous tissue, muscle	Occasional pain	None	150	2–3.9	300
74 F	N/A	Venous leg ulcer	Hypertension	None	Skin, subcutaneous tissue, muscle	Undermined wound bed, purulent exudate (xxx), continuous pain, malodor through dressing	None	150	1–2	150
76 F	N/A	Pressure ulcer	Diabetes, hypertension	BMI >30, smoking	Subcutaneous tissue	Biofilm, serous exudate (xx), occasional pain	None	130	Not stated	Not stated
73 M	N/A	Diabetic foot	Diabetes, PAD	None	Skin, subcutaneous tissue	Undermined wound bed, serous discharge, occasional pain, malodor following dressing removal	None	6	>1	3
64 F	N/A	Wound of unknown ethiology (susp. Insect bite)	None	None	Skin, subcutaneous tissue, muscle tendon	Biofilm, bloody exudate (xx), occasional pain	None	3	2–3.9	9
69 F	N/A	Pressure ulcer	Diabetes, PAD, hypertension, neuropathy	Smoking	Skin, subcutaneous tissue	Necrosis/gangrene, serous exudate (xx), pain during dressing change, malodor following dressing removal	*S. aureus*, E.coli, C. albicans	36	2–3.9	90
69 M	N/A	Diabetic foot	PAD	None	Skin	Biofilm, serous exudate (x), pain during dressing change	None	38.5	Not stated	Not stated
49 M	N/A	Venous leg ulcer	Diabetes, chronic venous insufficiency	BMI >30, smoking, alcoholism	Skin	Biofilm, serous exudate (x), occasional pain	None	2.25	Not stated	Not stated
78 F	N/A	Diabetic foot, defect after amputation of 2nd and 3rd toe	Diabetes, PAD, polyneuropathy, hypertension, renal failure	None	Skin, subcutaneous tissue, muscle	Biofilm, serous exudate (x), pain during dressing change	None	12	1–2	18
79 M	N/A	Heel pressure ulcer	Diabetes	Smoking	Subcutaneous tissue	Biofilm, serous exudate (xx), occasional pain	None	7.5	>1	4
62 M	N/A	Venous leg ulcer	PAD, hypertension	BMI >30	Skin	Biofilm, serous exudate (x)	Staph. epidirmidis, *E. coli*	6	>1	2
83 F	N/A	Venous leg ulcer	PAD	None	Skin	Undermined wound bed, purulent discharge, continuous pain, intense malodor	None	n/a	Not stated	Not stated
82 F	N/A	Venous leg ulcer	Varicose veins	BMI >30	Skin, subcutaneous tissue	Undermined wound bed, purulent discharge, continuous pain, intense malodor	None	104	>1	52
79 M	N/A	Heel pressure ulcer	Diabetes, PAD, polyneuropathy	None	Skin, subcutaneous tissue	Biofilm, serous exudate (xx), occasional pain	*S. aureus*, *E. coli*, C. albicans	7.5	>1	2
69 F	N/A	Venous leg ulcer	Alcoholic liver cirrhosis, chronic venous insufficiency	Smoking, alcoholism	Skin	Biofilm, occasional pain	*Serratia marcescens*, Staph.epidermidis	32	Not stated	Not stated
53 M	N/A	Venous leg ulcer	None	Smoking, alcoholism	Skin, subcutaneous tissue	Undermined wound bed, purulent discharge (xxx), occasional pain, malodor upon dressing removal	None	16	>1	8
72 M	N/A	Venous leg ulcer	Diabetes, PAD	BMI >30, smoking, alcoholism	Skin, subcutaneous tissue	Biofilm, serous exudate (xx) continuous pain	*E. coli*, C. albicans	27	>1	14
78 F	N/A	Pressure ulcer	Hypertension	None	Skin, subcutaneous tissue, muscle	Necrosis/gangrene, serous exudate (xx), occasional pain	None	6	2–3.9	18
77 F	N/A	Venous leg ulcer	PAD	BMI >30	Skin, subcutaneous tissue	Serous exudate, pain during dressing change, malodor upon dressing removal	None	10	>1	3
73 M	N/A	Pressure ulcer	Diabetes – insulin dependent, PAD	BMI >30	Skin, subcutaneous tissue, muscle, tendon	Necrosis/gangrene, purulent discharge, continuous pain, intense malodor	*Clostridium* spp.	25	2–3.9	75
74 M	N/A	Wound (traumatic)	None	None	Skin, subcutaneous tissue, muscle, tendon	Biofilm, serous exudate (xx), pain during dressing change	None	7.5	>1	4
84 F	N/A	Pressure ulcer	Diabetes	BMI >30	Skin, subcutaneous tissue	Undermined wound bed, serous exudate (xx), pain during dressing change, malodor upon dressing removal	None	225	>1	113
80 F	N/A	Venous leg ulcer	None	None	Skin, subcutaneous tissue	Undermined wound bed, purulent discharge, continuous pain	None	400	not stated	not stated
73 F	N/A	Osteomyelitis	Diabetes, Pseudoarthrosis tibiae congenita	None	Skin, bone	Undermined wound bed, purulent discharge, pain during dressing change, malodor upon dressing removal	MRSA	6	4–5.9	24
68 M	N/A	Ischemic Foot ulcer	Diabetes, PAD, ischemic foot	BMI >30	Skin, subcutaneous tissue	Undermined wound bed, purulent discharge (xxx), continuous pain, intense malodor	None	225	>1	180
65 F	N/A	Diabetic foot	Diabetes, PAD, hypertension	BMI >30	Skin, subcutaneous tissue	Necrosis/gangrene, serous exudate, continuous pain, malodor through dressing	None	20	1–2	20
61 M	N/A	Wound (other)	Dyspnea, chronic kidney disease, anemia, hypertension, hypothyreosis, arrhythmia	None	Skin, subcutaneous tissue	None	None	0.15	>1	0
66 F	N/A	Venous leg ulcer	PAD	Smoking	Skin, muscle	Undermined wound bed, necrosis/gangrene, pain during dressing change, malodor upon dressing removal	None	50	2–3.9	100
62 F	N/A	Pressure ulcer	None	BMI >30	Skin, subcutaneous tissue, muscle	Undermined wound bed, purulent discharge, malodor upon dressing removal	None	90	4–5.9	495
52 M	N/A	Diabetic foot	Diabetes	None	Skin, subcutaneous tissue	None	None	64	1–2	64
83 M	N/A	Pressure ulcer	None	None	Skin, subcutaneous tissue	Necrosis, purulent discharge, pain during dressing change, malodor upon dressing removal	*Proteus mirabilis*, *P. aeruginosa*	16	1–2	24
77 F	N/A	Venous leg ulcer	PAD	Smoking, alcoholism	Skin, subcutaneous tissue	Undermined wound bed, purulent discharge, occasional pain	None	72	1–2	108
82 F	N/A	Cancer (melanoma)	Cancer	None	Skin, muscle	Undermined wound bed, purulent discharge, continuous pain, intense malodor	None	50	0	0
78 F	N/A	Wound (traumatic)	Diabetes, PAD, hypertension, neuropathy	None	Skin	Occasional pain	None	60	not stated	not stated
66 M	N/A	Diabetic foot	Diabetes	BMI >30, smoking	Skin, subcutaneous tissue	None	None	16	>1	8
74 F	N/A	Venous leg ulcer	PAD	BMI >30, smoking	Skin, subcutaneous tissue	None	None	49	1–2	49
67 M	N/A	Diabetic foot	Diabetes, PAD	Smoking	Skin, subcutaneous tissue	Biofilm, serous exudate, pain during dressing change, malodor upon dressing removal	None	80	>1	32
52 F	N/A	Venous leg ulcer	Hypertension	BMI >30, smoking	Skin	Biofilm, serous exudate (xx), pain during dressing change	None	10.5	not stated	not stated
73 M	N/A	Pressure ulcer	PAD, hypertension	BMI >30	Skin, muscle	Necrosis/gangrene, purulent discharge, pain during dressing change, intense malodor	None	24.9	2–3.9	75
77 M	N/A	Stomic wound	Diabetes	BMI >30, smoking	Skin	None	None	49	>1	25
71 F	N/A	Stomic wound	None	BMI >30	Skin	Malodor upon dressing removal	None	64	>1	32
89 F	N/A	Venous leg ulcer	PAD	None	Skin, subcutaneous tissue	Undermined wound bed, purulent discharge, occasional pain, malodor upon dressing removal	None	70	not stated	not stated

### 3.5 Methods of use of DebriEcaSan Alfa

The most common method of use was soaking a piece of gaze or other material in the irrigation solution and leaving it in the wound for 10–20 min before proceeding with a dressing change. 211 respondents applied this method. The remaining users reported spraying, irrigating, or flushing the wound with DebriEcaSan Alfa before applying primary dressing, typically a gel. The reported exposure time ranged from 1 min to 3 h. The frequency of dressing changes ranged from 5-times a day to once a week. DebriEcaSan Alfa is typically used with barrier cream to protect the wound edges and other primary and secondary dressing. Additional interventions included surgical debridement, necrectomy, and larval therapy. Of the 239 patients, 77 were treated with intravenous and oral antibiotics.

### 3.6 Wound healing

#### 3.6.1 Affected tissues

**FIGURE 4:**
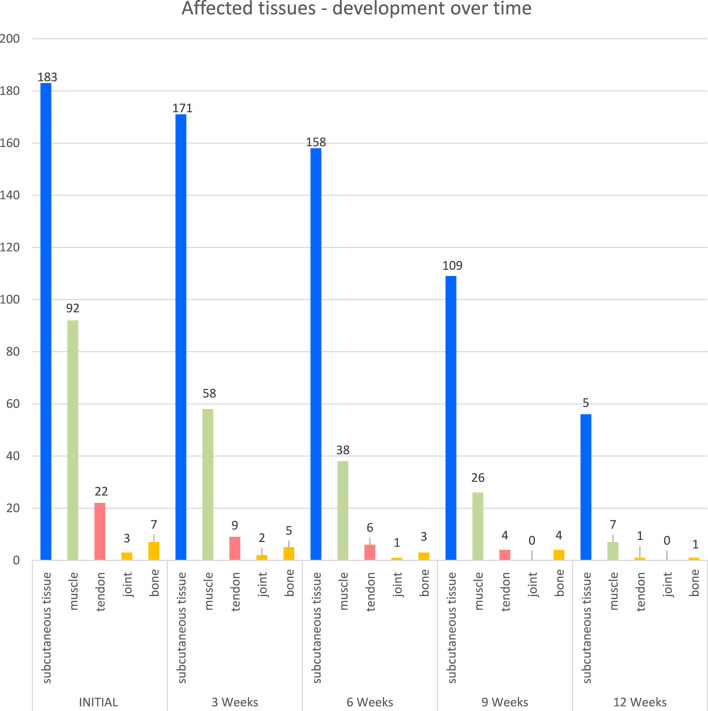
Affected tissues.

The number of patients with wounds affecting subcutaneous tissue steadily decreased from 183 at the initial examination to 171 at week 3, 158 at week 6, 109 at week 9, and 56 at week 12. Similarly, the number of wounds affecting muscle decreased from 92 at the initial examination to 58 at week 3, 38 at week 6, 26 at week 9, and 7 at week 12. There is a downward trend for wounds affecting the tendon from the initial 22 to 9 at week 3, 6 at week 6, 4 at week 9, and 1 at week 12. The number of wounds affecting joints and bones also decreased over time (see Figure 4).

#### 3.6.2 Symptoms of infection

##### 3.6.2.1 Symptoms of infection

**FIGURE 5:**
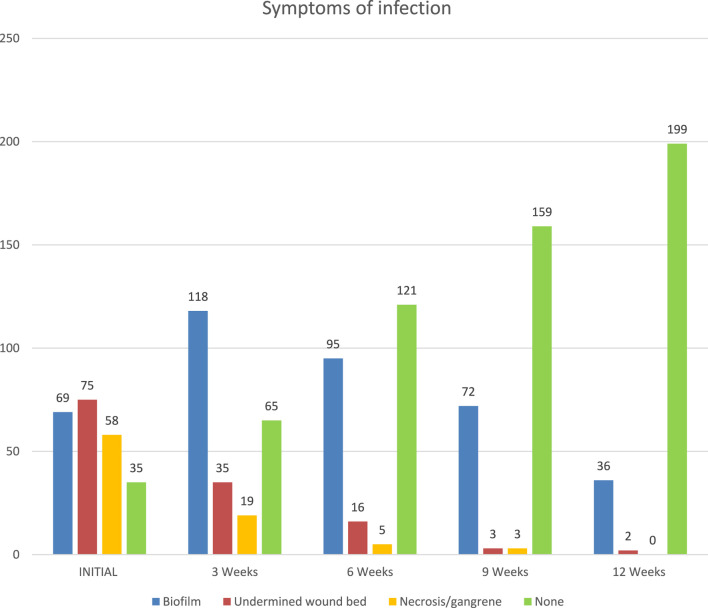
Symptoms of infection: biofilm, undermined wound bed, necrosis/gangrene.

The number of patients with wounds with biofilm increased by week 3 from 69 (29%) to 118 (50%). After this peak, there is a downward trend from week 3 to week 12. The number of wounds with undermined wound beds decreased from 75 (32%) at the initial examination to 15 (16%) at 6 weeks, 3 (1%) at 9 weeks, and 2 (1%) at 12 weeks. Similarly, the number of patients with necrotic, gangrenous wounds dropped from 58 (24%) at the initial examination to 19 (8%) at weeks, 5 (2%) at 6 weeks, 3 (1%) at 9 weeks, and 0 at 12 weeks. The number of patients with no symptoms of infection steadily grew from 35 (15%) at the initial examination to 65 (27%) at week 3, 121 (51%) at week 6, 159 (67%) at week 9, and 199 (84%) at week 12. A significant number of patients had infected wounds: 69 (29%) presented with biofilm, 75 (32%) had undermined wound beds, and 58 (24%) had wounds that were necrotic or gangrenous. The number of wounds with infection symptoms steadily decreased over the 12 weeks of treatment (see Figure 5). Of the 118 patients with biofilm at week 3, only 8 reported microbiological findings: *Bacteroides fragilis* (1), *Enterobacter cloacae*, (1), *Staphylococcus aureus* (3), *Proteus mirabilis* (1), and *Pseudomonas aeruginosa* (1).

##### 3.6.2.2 Exudate

**FIGURE 6:**
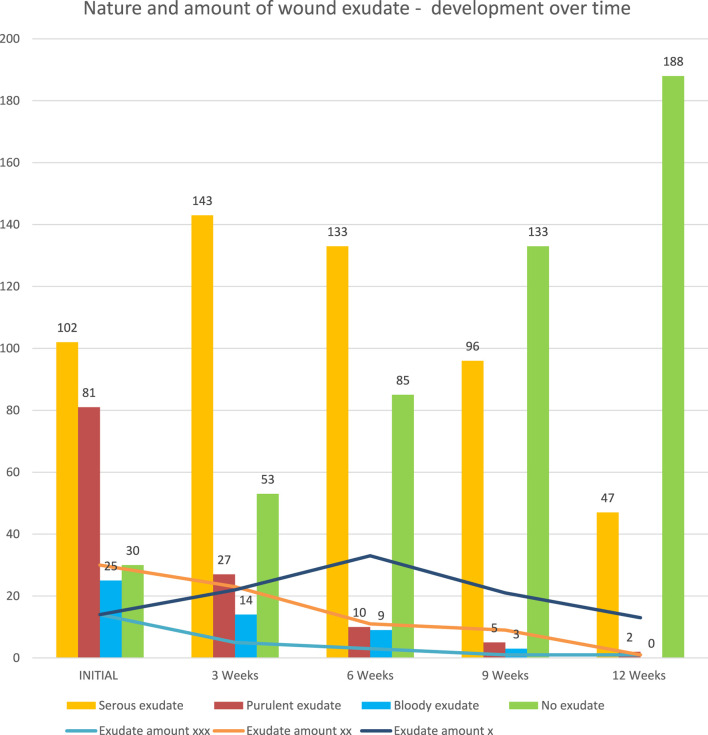
Nature and amount of wound exudate–development over time (graph).

The number of wounds with purulent, and bloody exudate decreased over time, partly changing to serous exudate, before clearing up completely. The number of wounds with no exudate increased from 30 (13%) during the initial examination to 188 (79%) at week 12. 102 (43%) patients presented with wounds secerning serous exudate; 25 (11%) had bloody exudate weeping from their wounds, and 81 (34%) showed purulent discharge. The number of wounds with purulent, and bloody exudate decreased over time, partly changing to serous exudate, before clearing up completely. The number of wounds with no exudate increased from 30 during the initial examination to 188 at week 12 (see Figure 6).

##### 3.6.2.3 Pain

**FIGURE 7:**
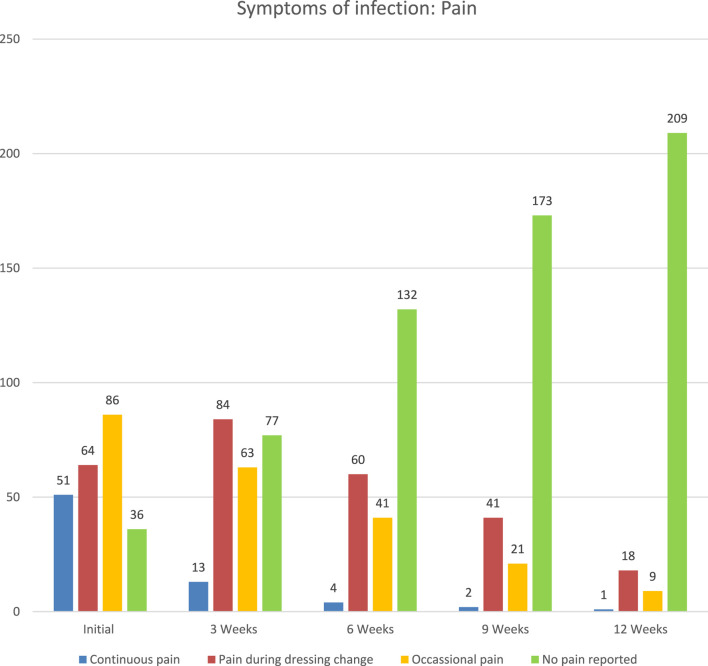
Reported pain over time.

The intensity and number of patients reporting pain decreased over the monitoring period. At the initial examination, only 36 (15%) patients reported no pain. This number increased to 77 (32%) at week 3, 132 (56%) at week 6, 173 (73%) at week 9, and 209 (88%) at week 12. Pain reported by patients decreased in intensity and numbers. At the initial examination, 36 (15%) patients reported no pain. This number increased to 77 (32%) at week 3, 132 (56%) at week 6, 173 (73%) at week 9, and 209 (88%) at week 12. At initial examination, 51 (22%) patients reported continuous pain, 64 (27%) experienced pain during dressing change, and 86 (36%) stated their pain was intermittent (See Figure 7).

##### 3.6.2.4 Malodor

**FIGURE 8:**
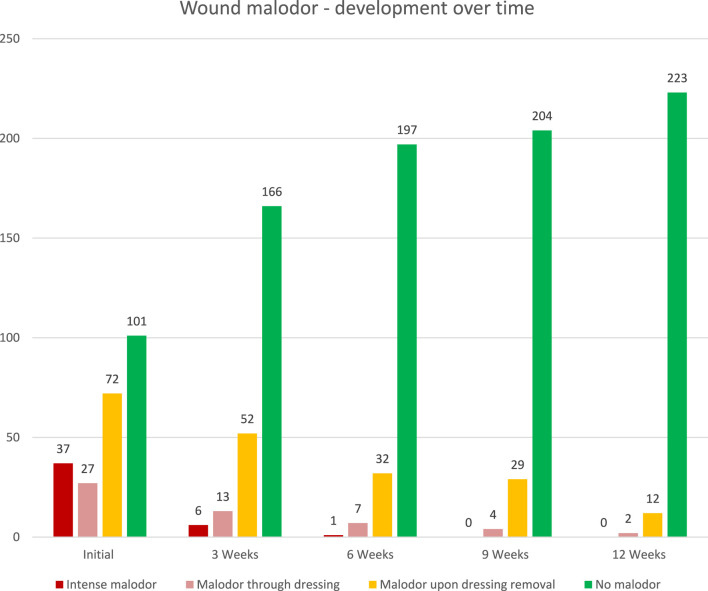
Wound malodor–development over time (graph).

Malodor was effectively eliminated within the first 3 weeks of treatment in the majority of patients. Intense malodor dropped from 37 (16%) at the initial examination to 6 (3%) at 3 weeks and 1 at 6 weeks. Wounds without malodor increased from 101 (43%) at the initial examination to 166 (70%) at week 3, 197 (83%) at week 6, 204 (86%) at week 9, and 223 (94%) at week 12. Initially, intense malodor affected 37 (16%) patients. An additional 27 (11%) patients reported malodor through dressing and 72 (30%) experienced malodor upon dressing removal. Malodor was effectively eliminated within the first 3 weeks of treatment in the majority of patients. The number of patients whose wounds expressed intense malodor dropped from 37 at the initial examination to 6 at 3 weeks and 1 at 6 weeks. The number of patients with wounds without malodor increased from 101 at the initial examination to 166 at week 3, 197 at week 6, 204 at week 9, and 223 at week 12 (See Figure 8).

#### 3.6.3 Wound microbiology

The majority of patients (179 out of 237) did not have any wound microbiology performed. The pathogens isolated from the 60 tested patients included *S. aureus*, *Staphylococcus haemolyticus*, *Staphylococcus epidermidis*, MRSA, MLSB, *Streptococcus dysgalactiae*, *Streptococcus* Beta-hemolytic group C, *Escherichia coli*, *Alcaligenes faecalis*, *Enterococcus cloacae*, *Enterococcus faecalis*, *P. mirabilis*, *Proteus vulgaris*, *P. aeruginosa*, *Morganella* spp., *Klebsiella pneumoniae*, *Klebsiella oxytoca*, *Serratia marcescens*, *Serratia odorifera*, *Clostridium Hathewayi*, *Clostridium* spp., *Corynebacterium Striatum*, and *Candida albicans*.

13 patients were tested in week three. The bacterial isolates included *E. cloacae* (1), *S. aureus* (4), *P. aeruginosa* (4), *Streptococcus haemolyticus* (1), *K. pneumoniae* (1), *P. mirabilis* (1), *B. fragilis* (1) and *Corynebacterium striatum* (1). Isolates cultivated in week 6 included *S. aureus* (2), *P. aeruginosa* (3), *E. coli* (2), and *K. pneumoniae* (1). Bacterial isolates from week 9 included *Escheria coli* (2), *Staphylococcus* *cohnii* (1), and *Enterobacter faecalis* (1). A combined infection caused by *Staphyloccocus capitis* and *B. fragilis* was detected in one patient in week 12.

#### 3.6.4 Wound healing: wound size and wound closure

##### 3.6.4.1 Granulation and epithelization

The progress of granulation and epithelization over the course of treatment shows steady increase in granulation tissue and epithelization. No hypergranulation was observed (Table 7).

**TABLE 7:** Wound granulation and epithelization–development over time.

	Initial	%	3W	%	6W	%	9W	%	12W	%
Granulation										
Granulation 0%	192	81%	48	20%	40	17%	80	34%	144	61%
Granulation 25%	31	13%	100	42%	57	24%	26	11%	10	4%
Granulation 50%	10	4%	63	27%	94	40%	62	26%	33	14%
Granulation 75%	1	0%	18	8%	18	8%	16	7%	9	4%
Granulation 100%	3	1%	8	3%	28	12%	53	22%	41	17%
Hypergranulation	0	0%	0	0%	0	0%	0	0%	0	0%
Epithelization										
Epithelization 0%	229	97%	125	53%	64	27%	76	32%	134	57%
Epithelization 25%	6	3%	78	33%	80	34%	51	22%	25	11%
Epithelization 50%	1	0%	26	11%	63	27%	58	24%	35	15%
Epithelization 75%	0	0%	3	1%	18	8%	12	5%	13	5%
Epithelization 100%	1	0%	5	2%	12	5%	40	17%	30	13%

##### 3.6.4.2 Wound size and depth

The wounds are routinely measured as part of standard treatment protocols. However, the methodology may differ from one facility to another. Some healthcare establishments routinely take photographs to document progress. A significant number of patients presented with large and deep wounds. At the initial presentation, 85 (36%) patients presented with wounds that were larger than 50 cm^2^, of which 8 (3%) had wounds larger than 500 cm^2^ and 16 (7%) between 200 and 499 cm^2^ (see Figure 7). A marked reduction in wound surface area size was observed in all wound size categories (see Table 8). 24 patients presented with large, deep and complex wounds. The healing times and outcomes reflect the nature and origin of the wounds, underlying disease and complications. This cohort illustrates the typical challenges experienced when measuring outcomes in wound healing (Table 9). Wound depth was stratified into ranges 0 (superficial), <1 cm, 1-1.9 cm, 2-3.9 cm, 4-5.9 cm, and >6 cm. Wound depth gradually decreased over the course of 12 weeks even in the most complex patients (Table 10). 19 (8%) patients healed by end of week 6; and 66 (28%) healed by week 9. 130 (55%) patients were considered healed by week 12. 23 (10%) patients were transferred to a different facility, 9 (4%) underwent surgery, 7 (3%) had treatment changed, and 5 (2%) died of their underlying disease. 63 (27%) patients were still healing at week 12 (see Table 11).

**TABLE 8:** Wound surface area size: development over time.

Wound size	Initial	%	3W	%	6W	%	9W	%	12W	%
0	0	0%	0	0%	0	0%	72	30%	121	51%
<4.9 cm^2^	20	8%	55	23%	76	32%	54	23%	40	17%
5–9.9 cm^2^	33	14%	29	12%	30	13%	19	8%	9	4%
10–19.9 cm^2^	33	14%	27	11%	21	9%	18	8%	9	4%
20–29.9 cm	29	12%	26	11%	19	8%	11	5%	8	3%
30–39.9 cm^2^	18	8%	21	9%	12	5%	2	1%	4	2%
40–49.9 cm^2^	16	7%	7	3%	4	2%	10	4%	4	2%
50–99 cm^2^	31	13%	25	11%	22	9%	17	7%	4	2%
100–199 cm^2^	30	13%	26	11%	21	9%	9	4%	5	2%
200–499 cm^2^	16	7%	10	4%	8	3%	4	2%	2	1%
>500 cm^2^	8	3%	8	3%	5	2%	1	0%	0	0%
not stated	3	1%	3	1%	19	8%	20	8%	31	13%

**TABLE 9:** Overview of wound characteristics and outcomes of patients with large wounds.

Patient	Basic diagnosis	Comorbidities and risk factors	Wound characteristics (initial) Location Affected structures Infection Microbiology	Time lag	Initial wound size Area Depth Volume	Treatment	Outcome
69 F	Erysipelas	Obesity	Lower limb Subcutaneous tissue Biofilm, serous exudate Occasional pain	2 days	A: 900 cm^2^ D: not stated V: not stated	Sterile gauze soaked in DebriEcaSan Alfa for 15 min; dressing change: twice a day; DebriEcaSan aquagel + Lomateul + sterile dressing; other interventions: limb positioning; antibiotics: Penicillin 7days i.v., Dalacin i.v. 7 days	Healed within 12 weeks
74 M	Venous leg ulcer	None	Lower limb Subcutaneous tissue Purulent discharge Occasional pain Intense malodor	136 days	A: 700 cm^2^ D: 2 cm V: 1,400 cm^3^	Sterile gauze soaked in DebriEcaSan Alfa for 15 min; dressing change: every other day; dressing: DES aquagel + secondary dressing; compression dressing	Healed within 20 weeks
55 M	Burn, 3rd degree	Homelessness, anoxic brain damage, obesity, smoking, alcoholism	Torso/pelvis Subcutaneous tissue Undermined wound bed Serous exudate Continuous pain Malodor upon removal of dressing	2 months	A: 600 cm^2^ D: 1 cm V: 600 cm^3^	Spraying the wound with DebriEcaSan Alfa 10 min; dressing change: daily; dressing: Xeroform, sterile gauze, omuifix; surrounding skin: ZinOxid	Status at 12 weeks: still healing, still hospitalized
58 M	Phlegmon Erysipelas	pulmonary hypertension, congestive right heart failure, heavy smoker	Lower limb Muscle, tendon Necrosis/gangrene Bloody exudate Pain during dressing change Intense malodor MRSA, *P. aeruginosa*	2 months	A: 2,400 cm^2^ D: 3 cm V: 7,200 cm^3^	Sterile gauze soaked in DebriEcaSan Alfa for 15 min; dressing change: every other day; dressing: tulle gras, sterile gauze; compression dressing; other interventions: necrectomy, debridement, analgesia p.o.+i.m., larval therapy; antibiotics: Ciplox 500 14 days, Penicillin 20 days, Dalacin 43 days	Status at 12 weeks: Transferred to surgery. Vacuum therapy, skin grafts
59 F	Radiation dermatitis (prophylaxis)	Cancer	Lower limb Skin Infection: none	2 months	A: 1,600 cm^2^ D: surface V: n/a	Sterile gauze soaked in DebriEcaSan Alfa for 20 min; dressing change: weekly; dressing: Meditel FLM; surrounding skin: linola radioderm	At 9 weeks change of treatment. Burning sensation during application of DebriEcaSan Alfa Wound size unchanged at 6 weeks
45 F	Radiation dermatitis (prophylaxis)	Cancer	Torso/pelvis Skin Infection: none	Not available	A: 900 cm^2^ D: surface V: n/a	Sterile gauze soaked in DebriEcaSan Alfa for 20 min; dressing change every other day; dressing: mepitel film; surrounding skin: linola radioderm	Radiation treatment completed, patient transferred
60 F	Radiation dermatitis (prophylaxis)	Cancer	Torso/pelvis Skin Infection: none	Not available	A: 4,900 cm^2^ D: surface V: n/a	Sterile gauze soaked in DebriEcaSan Alfa for 10–15 min; dressing change: daily; dressing: mepitel film	Radiation treatment completed, patient transferred
69 F	Leg ulcer, combined etiology	Polymorbid, obesity	Torso/pelvis Subcutaneous tissue, muscle Undermined wound bed Purulent discharge Continuous pain Malodor upon removal of dressing	Not available	A: 900 cm^2^ D: not stated V: not stated	Sterile gauze soaked in DebriEcaSan Alfa for 20 min; dressing change: daily; dressing: DebriEcaSan aquagel + Lomatuel + sterile dressing; other interventions: limb positioning; antibiotics: Amoksiklav i.v. 7 days	Discharged at 6 weeks to finish healing, wound size at discharge was 20 × 20 cm (400 cm^2^)
52 M	Venous leg ulcer	None	Lower limb subcutaneous tissue biofilm, serous exudate, occasional pain, malodor after dressing removal G- (Unspecified)	more than 10 years	A: 300 cm D: 1.5 cm^2^ V: 450 cm^3^	Sterile gauze soaked in DebriEcaSan Alfa for 3 h; dressing change: daily; dressing: Boragent ointment + Zetuvit; compression dressing; other interventions: necrectomy	Status at 12 weeks: wound size 280 cm^2^, depth 1.5 cm. Discharged, continues treatment at home
53 M	Wound (traumatic)	Chronic bronchitis, casus socialis, smoking, alcoholism	Lower limb subcutaneous tissue necrosis, bloody and purulent exudate, continuous pain, malodor through dressing	10 days	A: 260 cm D: 0.3 cm^2^ V: 78 cm^3^	Sterile gauze soaked in DebriEcaSan Alfa for 15 min; dressing change: daily; dressing: telfa, uliwazel, bandage; other interventions: necrectomy; antibiotics: amoksiklav 625g/8d.,ciplox 250g/5d., imtizol 250g/8d	Status at 3 weeks: wound size 150 cm^2^; patient transferred
54 F	Acute uremic syndrome	scleroderma	Torso/pelvis subcutaneous tissue biofilm, purulent discharge, continuous pain, none MRSA	13 days	A: 400 cm D: 0.1 cm^2^ V: 40 cm^3^	Sterile gauze soaked in DebriEcaSan Alfa for 20 min; dressing change: every other day; dressing: HyalEcaSan + Telfa	Healed at 6 weeks
59 M	Wound (other)	cancer, obesity	Torso/pelvis subcutaneous tissue, muscle necrosis, serous exudate, occasional pain, intense malodor	Same day	A: 400 cm D: 15 cm^2^ V: 6,000 cm^3^	Irrigation with DebriEcaSan Alfa; duration not stated; dressing change: daily; dressing: DebriEcaSan aquagel, Xeroform, sterile dressing thorough hygiene of wound surrounding for 6 days	Status at 12 weeks: worsening of primary disease, patient transferred
62 F	Venous leg ulcer	Varicose veins, leg edema, obesity	Lower limb subcutaneous tissue biofilm, serous exudate xxx, pain during dressing change, malodor after dressing removal	121 days	A: 240 cm D: 0.5 cm^2^ V: 120 cm^3^	Sterile gauze soaked in DebriEcaSan Alfa; duration not stated; dressing change: 3 times a week; dressing: Vliwaktiv + Resposorb; compression dressing Lenkideal; other interventions: debridement of wound bed	Status at 12 weeks Wound size 15 cm^2^ Healed, 13+ weeks
63 M	Venous leg ulcer	Diabetes, peripheral artery disease	Lower limb subcutaneous tissue, muscle necrosis/gangrene, bloody exudate, occasional pain, intense malodor MRSA, *P. aeruginosa*	10 months	A: 400 cm D: 2.5 cm^2^ V: 1,000 cm^3^	Sterile gauze soaked in DebriEcaSan Alfa for 15 min; dressing change: daily; dressing: DES aquagel + Telfa; compression dressing; surrounding skin: barrier cream; antibiotics: Augmentin	At 12 weeks: treatment continues, still healing Wound size at 12 weeks: 13 × 13 cm (169 cm^2^)
64 M	Venous leg ulcer	Diabetes, obesity	Lower limb subcutaneous tissue undermined wound bed, serous exudate, pain during dressing change, malodor after dressing removal	More than 10 years	A: 300 cm D: 2 cm^2^ V: 600 cm^3^	Sterile gauze soaked in DebriEcaSan Alfa; duration not stated; dressing change: daily; dressing: Vliwazel, Xeroform, HyalEcaSan; patient refused compression dressing; surrounding skin: ZinOxid; antibiotics: 7 days	Healed at 9 weeks
67 F	Venous leg ulcer	Heart failure, asthma, obesity	Lower limb subcutaneous tissue undermined wound bed, purulent discharge, occasional pain, malodor after dressing removal	6 days	A: 400 cm D: 0.5 cm^2^ V: 200 cm^3^	Sterile gauze soaked in DebriEcaSan Alfa for 20 min; dressing change: every other day; dressing: DES aquagel, Xeroform, Zetuvit; compression dressing	Discharged; wound size at 9 weeks 225 cm^2^
68 M	Ischaemic foot ulcer	Diabetes, peripheral artery disease, ischemic foot, peripheral artery disease, obesity	Lower limb subcutaneous tissue undermined wound bed, purulent discharge xxx, continuous pain, intense malodor	Not available	A: 225 cm D: 0.8 cm^2^ V: 180 cm^3^	Sterile gauze soaked in DebriEcaSan Alfa; duration not stated; dressing change: 3 times a week; dressing: tulle grass + Vliwazel	Still healing, wound size at 12 weeks 10 × 10 cm (100 cm^2^)
73 F	Venous leg ulcer	Diabetes	Lower limb subcutaneous tissue undermined wound bed, bloody exudate xxx, pain during dressing change, malodor after dressing removal	More than 5 years	A: 200 cm D: 0.5 cm^2^ V: 100 cm^3^	Sterile gauze soaked in DebriEcaSan Alfa for 10 min; dressing change: every other day; dressing: DebriEcaSan aquagel, tulle gras, sterile gauze + zetuvit; compression dressing	Discharged at 5 weeks. Wound size 162 cm^2^
74 F	Wound (surgical)	Diabetes, peripheral artery disease	Torso/pelvis subcutaneous tissue, muscle undermined wound bed, bloody exudate, occasional pain, malodor through dressing	17 days	A: 200 cm D: 10 cm^2^ V: 2000 cm^3^	Sterile gauze soaked in DebriEcaSan Alfa for 15 min; dressing change: daily; dressing: DES aquagel + Xeroform; surrounding skin: barrier cream	Still healing, wound size at 12 weeks 60 cm^2^
79 M	Wound (other)	Cancer	Lower limb subcutaneous tissue, muscle undermined wound bed, purulent discharge, occasional pain, intense malodor	same day	A: 250 cm D: 5 cm^2^ V: 1,250 cm^3^	Drain inserted in gauze, regularly irrigated with DebriEcaSan Alfa; duration not stated; dressing change: daily; dressing: packing the wound with sterile dressing + sterile secondary dressing; compression dressing; surrounding skin: Cavilon; antibiotics: 7 days	Healed at 12 weeks
80 F	Venous leg ulcer	Peripheral artery disease	Lower limb subcutaneous tissue undermined wound bed, purulent discharge, continuous pain, malodor after dressing removal	Not available	A: 400 cm D: not stated V: not stated	Sterile gauze soaked in DebriEcaSan Alfa for 20 min; dressing change: daily; dressing: DebriEcaSan aquagel + sterile dressing, 1 week Exufiber Ag; protection of interdigital area; antibiotics: Meronem 8 days	Discharged at 6 weeks, wound size at 9 weeks: 168 cm^2^
81 F	Venous leg ulcer	Obesity subcutaneous tissue, muscle	Lower limb subcutaneous tissue biofilm, serous exudate, occasional pain, malodor after dressing removal MLSB	Several months	A: 234 cm D: 0.4 cm^2^ V: 94 cm^3^	Sterile gauze soaked in DebriEcaSan Alfa for 20 min; dressing change: every other day; dressing: Xeroform, Zetuvit + DES aquagel; compression dressing; surrounding skin: ZinOxid; antibiotics: according to sensitivity	Still healing, wound size at 12 weeks 70 cm^2^
82 F	Venous leg ulcer	Diabetes, obesity	Lower limb subcutaneous tissue biofilm, serous exudate, pain during dressing change, malodor after dressing removal *P. aeruginosa*	64	A: 323 cm D: 0.3 cm^2^ V: 97 cm^3^	Sterile gauze soaked in DebriEcaSan Alfa for 10 min; dressing change: daily; dressing: Lomattul H, gauze compress, AB kompres	Discharged at 13 weeks, wound size at 1 week: 255 cm^2^
84 F	Pressure ulcer	Diabetes, obesity	Torso/pelvis subcutaneous tissue undermined wound bed, serous exudate xx, pain during dressing change, malodor after dressing removal	Not available	A: 225 cm D: 0.5 cm^2^ V: 113 cm^3^	Sterile gauze soaked in DebriEcaSan Alfa for 15 min; dressing change: daily; dressing: DebriEcaSan aquagel, Telfa, Melgisorb Ag for the first 5 days; surrounding skin: okolí ZinOxid	Discharged at 10 weeks, wound size not known, 100% epithelization

**TABLE 10:** Wound depth.

Wound depth	Initial	%	3W	%	6W	%	9W	%	12W	%
0	0	0%	0	0%	0	0%	67	28%	140	59%
<1 cm	114	48%	119	50%	145	61%	94	40%	55	23%
1–1.9 cm	42	18%	42	18%	38	16%	27	11%	4	2%
2–3.9 cm	39	16%	30	13%	20	8%	14	6%	6	3%
4–5.9 cm	13	5%	10	4%	8	3%	4	2%	2	1%
>6 cm	8	3%	7	3%	5	2%	2	1%	1	0%
not stated	21	9%	29	12%	21	9%	29	12%	29	12%

**TABLE 11:** Wound healing.

Healing	Initial	%	3W	%	6W	%	9W	%	12W	%
Healed	0	0%	0	0%	19	8%	67	28%	130	55%
Not healed	237	100%	237	100%	214	90%	155	65%	63	27%
Transferred	0	0%	0	0%	4	2%	10	4%	23	10%
Change of treatment	0	0%	0	0%	0	0%	1	0%	7	3%
Died	0	0%	0	0%	0	0%	2	1%	5	2%
Surgery	0	0%	0	0%	0	0%	3	1%	9	4%

##### 3.6.4.3 Healed wounds

Healing time is one of the most important clinical outcomes in wound care. However, an accurate reading is difficult to obtain because the patients typically only stay in the same facility for part of the duration of their treatment. Moreover, only a minority of wounds are the primary reason for hospitalization but rather a comorbidity or a complication of treatment. In this study, the wounds were marked by healthcare staff as healed either upon complete closure of the wound, where possible, or at discharge from the hospital to a different type of facility or home care when the wound no longer required advanced care. This inconsistency causes a discrepancy between declared wound size and healing status. Hence, wounds that are almost healed at the point of transfer or dismissal are considered healed. More accurate readings can only be obtained from health data across multiple care systems.

##### 3.6.4.4 Case report

**FIGURE 9:**
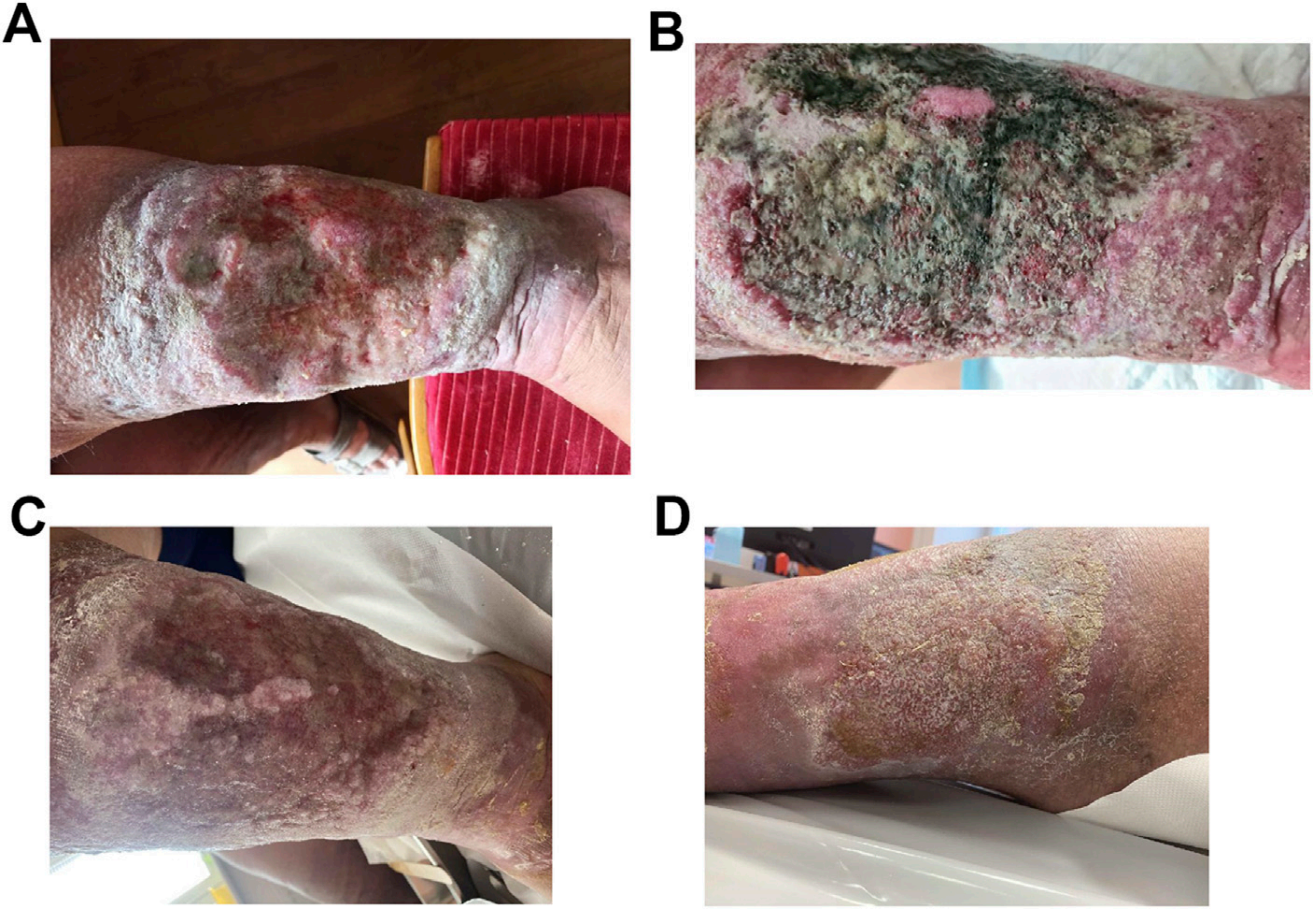
**(A)** Venous leg ulcer in a 64-year-old male patient (18 July 2022). **(B)** Infected venous leg ulcer (22 August 2022). **(C)** Significantly improved venous leg ulcer (19 September 2022). **(D)** Healed venous leg ulcer (21 November 2022).

A case report of a 64-year-old obese, diabetic male with venous leg ulcer demonstrates how a large defect (20 × 15 cm) healed over the course of 4 months with daily treatment with DebriEcaSan Alfa, DebriEcaSan aquagel, Xeroform, and compression dressing Vliwazel (see Figures 9A–D).

#### 3.6.5 Venous leg ulcer: A case report

A 64-year-old male patient, S.P., was treated at the Dobrovskeho Polyclinic, 1st surgical clinic, Brno, for a venous leg ulcer. The patient’s medical history included obesity, diabetes, and limited mobility of the lower extremities. The patient presented with severe limb edema but was unwilling to use a compression bandage. The patient was able to walk a short distance without aid but breathless. Difficulty breathing was also apparent at rest during prolonged conversation. The patient was not following the diabetic diet he was prescribed. The patient was adequately hydrated, calm, oriented, had a good memory, and was communicating adequately. The patient reported intermittent pain at the site of ulceration and is currently without pain medication.

The patient presented at the surgery with a venous leg ulcer on the lateral side of his left lower limb, size 20 × 15 cm, with an undermined wound bed without signs of ascendent infection. Sterile gauze soaked with wound irrigation solution DebriEcaSan Alfa was applied into the wound for 15–20 min, followed by primary gel dressing DebriEcaSan aquagel, a petrolatum-based fine mesh gauze containing 3% bismuth tribromophenate Xeroform, and compression dressing Vliwazel. The dressing was changed every 24 h.

A month later, the venous leg ulcer, size 20 × 15 cm, with sweet malodor and signs of infection. Cultivation revealed *P. mirabilis*. In addition to the existing treatment protocol, the patient received systemic antibiotics.

Two months after the initial presentation, the ulcer showed marked improvement, with a reduction in size (both surface area and depth) to 10 × 5 cm and minimal secretion. The wound shows granulation and epithelization progressing from the edges. The treatment protocol includes sterile gauze soaked in DebriEcaSan Alfa applied to the wound for 15–20 min, followed by DebriEcaSan aquagel, Xeroform dressing, and compression dressing Vliwazel. The dressing was changed every 24 h.

Four months after the initial presentation, the ulcer healed completely.

#### 3.6.6 Complications, adverse events

In total, ten patients experienced complications as reported on the form, most of which related to the underlying condition. Three patients experienced adverse events that have a plausible causal relationship to DebriEcaSan Alfa: maceration of wound edges (64 M with pressure ulcer), burning and itching (59 F with radiation dermatitis), and burning and stinging (70 F with venous leg ulcer).

## 4 Discussion

The use of antiseptics for wound irrigation remains controversial and no authoritative recommendation currently exists for the use of specific solutions and methods for the irrigation of pressure ulcers (European Pressure Ulcer Advisory Panel, National Pressure Injury Advisory Panel and Pan Pacific Pressure Injury Alliance, 2019), infected leg ulcers (National Institute for Health and Care Excellence, 2020), and diabetic foot ulcers (Saeg et al., 2021; Eriksson et al., 2022; Senneville et al., 2024).

In this PMCF study, we found that superoxidized solution DebriEcaSan Alfa is safe and effective in the treatment of acute and chronic wounds, leading to wound size reduction, improved granulation and epithelization, and decrease in microbial load, leading to the reduction of malodor and pain.

As documented in numerous *in vitro* studies, various brands of superoxidized solutions are effective against a number of aerobic, facultatively aerobic, and anaerobic bacteria, viruses, bacterial spores, bacteriophages and fungi (Cloete et al., 2009; Rossi-Fedele et al., 2010; Thorn et al., 2011; Ono et al., 2012; Mena-Mendivil et al., 2013; Torres-Capetillo et al., 2013; Gunaydin et al., 2014; Sakarya et al., 2014; Armstrong et al., 2015; Gray et al., 2016; Al-Mualla et al., 2018; Herruzo and Herruzo, 2020; Aranke et al., 2021; Jimenez-Gonzalez et al., 2021). They also show good efficacy against biofilms (Gold et al., 2020; Sakarya et al., 2014; Armstrong et al., 2015; Al-Mualla et al., 2018; Cai et al., 2018; Harriott et al., 2019; Schwarzer et al., 2019; Savadkouhi et al., 2021; Salisbury and Percival, 2019). DebriEcaSan Alfa demonstrated similar microbicidal properties *in vitro*, including *P. aeruginosa*, *S. aureus*, *Enterococcus* hirae, and E.coli K12, *Candida* albicans, Aspergillus brasiliensis (niger), *Mycobacterium avium* and *Mycobacterium* terrae. The majority of evidence of the antimicrobial efficacy of superoxidized solutions comes from *in-vitro* studies. Due to this lack of evidence from human *in-vivo* studies, no recommendations exist to support one irrigation solution over the others using clinical endpoints, such as time to heal, reduction of wound bioburden, elimination of infection, or the rate of complications. Superoxidized solutions have minimal to low cytotoxicity and are widely recognized as non-sensitizing and non-irritating (Landa-Solis et al., 2005; Gutiérrez, 2006; Gonzalez-Espinosa et al., 2007; le Duc et al., 2007; Gomi et al., 2010; Ortega-Pena et al., 2017; Salisbury and Percival, 2019; Severing et al., 2019). DebriEcaSan Alfa was proven to be non-cytotoxic and non-irritating.

The patient demographics included in the study was a representative sample of wound patients in regard to age, sex, basic diagnosis, comorbidities, and risk factors. The majority of the patients included in the study were adults, older adults and the elderly. Of the 237 patients treated with DebriEcaSan Alfa, only a small minority were younger than 45 years of age. Both sexes are equally represented. The most common diagnoses were venous leg ulcer (91; 38%), pressure ulcer (41; 17%), diabetic foot ulcer (28; 12%), and traumatic wound (18; 8%).

Diabetic patients are more likely to develop polymicrobial wound infections due to impaired leucocyte function and suboptimal inflammatory response (Burgess et al., 2021), leading to poor formation of granulation tissue and delayed wound healing. Obesity adversely affects healing through poor vascularization of adipose tissue, oxidative stress, abnormalities in the function of immune mediators, and nutritional deficiencies (Pierpont et al., 2014). Unsurprisingly, diabetes (90; 38%), obesity (99; 42%), peripheral artery disease (79; 33%), and tobacco use (73; 31%) turned out to be the most frequently cited conditions in non-healing wounds. This patient risk profile is consistent with data reported from literature (Pokorna, 2017).

The wound characteristics varied greatly in terms of wound severity, size, chronicity, and the presence of infection. The wounds that were older than 3 months at the time of presentation tended to be complex, large, deep, and with symptoms of infection.

The severity and size of the wounds decrease steadily during the 12 weeks of treatment, with improvement apparent in all aspects of wound healing, from tissues affected to symptoms of infection, malodor, and pain. Somewhat unexpectedly, DebriEcaSan Alfa was typically used not as an irrigation solution but as a poultice. Healthcare staff left material soaked in the solution for 10–20 min before proceeding with a dressing change, in an apparent attempt to utilize antimicrobial function of DebriEcaSan Alfa to combat wound infection. This fact only became apparent due to the survey questions that prompted the respondents to describe how they use the product in clinical practice, without making any suggestions regarding its correct use. None of the current clinical guidelines recommends poultice as the preferred method of application. About a third (77 out of 239 patients) received oral or intravenous antibiotics. Additional interventions included surgical debridement, necrectomy, and larval therapy. As documented by Pokorna (2017), the paucity of data available through official reporting systems makes it impossible to establish baseline. The overall severity of a case is defined by detailed characteristics and grading of the wound itself, as well as the patient’s comorbid conditions, and their ability for self-care. The severity of a case directly impacts the expected healing times, the rate and nature of complications, and healing outcomes. This level of detail cannot be obtained from existing databases for comparison. Without such baseline, it is impossible to tell how specific interventions perform in comparison to alternative treatment options.

Pain and malodor are measures very important to patients, yet, available literature remains largely silent on these endpoints. No data on the duration of treatment and cost of treatment is available in national registries. This dataset provides an important benchmark needed for comparison in future studies.

Clinical research in wound care faces specific challenges. Typically, patients with extensive medical histories present with chronic, complex wounds that require interventions that are highly visible and difficult to blind. Different staff members treat the patients over an extended period, and often across multiple care settings. Treatment is often modified in response to the stage of healing and emerging complications, making each wound an experiment. A chronic wound is often not a primary reason for hospitalization but a comorbidity or, in the case of pressure ulcers, a complication of hospitalization. Moreover, a treatment protocol is often modified upon transfer to a different healthcare setting, such as discharge from acute care to a long-term care facility, outpatient, or home care. Additional limitations in medical device studies are of a regulatory nature.

The most common experimental and non-experimental designs used in wound care (Stephenson, 2022), include parallel randomized controlled trials and cross-over trials. Cluster trials assign a specific treatment protocol to all patients within a specific facility. Randomized controlled trials are relatively rare in the field of wound care due to challenges with blinding and appropriate sample sizes. Additional options include quasi-experimental designs, cohort studies, and case-control studies. Even observational studies can provide a high level of evidence. The most common design in wound care is a single-sample study, also called a “pre-post” or “paired” design, where the patients act as their own control. Here, the researchers collect data points pre- and post-intervention, such as wound pH and change of wound size from baseline (Stephenson, 2022).

The Food and Drug Administration (FDA) issued guidance on generating real-world evidence to support regulatory submissions for medical devices. For example, RWE can be used as a historical control, a prior in a Bayesian trial. RWE can also serve as a control group or provide evidence for expanding the device labeling to include additional indications for use or to add new information on safety and effectiveness (FDA, 2017).

This PMCF study offers valuable insight into the real-world use of wound irrigation solution DebriEcaSan Alfa (NewWater Meaning s.r.o.) in the Czech Republic. The data show that the patients who present with chronic wounds tend to suffer from multitude of comorbidities and risk factors that interfere with the healing process. The wounds also tend to be large, deep, complex, and often infected at the initial examination. A significant number of patients presented with wounds that were many months and even years old. DebriEcaSan Alfa is typically used on a soaked sterile gauze and applied for several minutes to increase its antimicrobial effect as opposed to simple irrigation as suggested by the manufacturer. This practice observed in clinical settings emphasizes the importance of biological compatibility, especially low cytotoxicity in combination with broad antimicrobial activity.

The observed clinical effect seems intuitively favorable. However, there is no objective baseline to compare the results to, as typical healing times in a comparable population are not accessible. No single standard of care exists in the treatment of chronic wounds, and significant variability in practices exists across the health system.

In the future, adequately designed and powered studies are needed to produce sufficient quality of evidence to provide confident recommendations in the product used and methods employed in wound irrigation.

## Data Availability

The original contributions presented in the study are included in the article/Supplementary Material, further inquiries can be directed to the corresponding author.
