# Supplementary material for: The safety and efficacy of neutral electrolyzed water solution for wound irrigation: post-market clinical follow-up study
Source: Front Drug Saf Regul. 2025 Jan 16;4:1402684. doi: 10.3389/fdsfr.2024.1402684 (PMC12443096; doi:10.3389/fdsfr.2024.1402684)
Supplement: Supplementary file 1 [file Table1.docx]

Supplementary Material

## Supplementary Figure 1 – Survey form questions included in the PMCF.

The survey questions below were distributed to both inpatient and outpatient healthcare facilities and nursing homes in the Czech Republic that use DebriEcaSan Alfa as part of their standard treatment protocol.

| **Administrative particulars:** | |
| --- | --- |
| Form number | NWM ID number |
| Name and job function of the evaluator |  |
| Name and address of the facility |  |
| Product(s) used | DebriEcaSan Alfa  DebriEcaSan aquagel  DebriEcaSan hyal plus  HyalEcaSan |
|  |  |
| **Patient characteristics** | |
| Patient ID code | NWM ID number |
| Date of first examination |  |
| Date when the wound first appeared |  |
| Age |  |
| Sex | M/F |
| Basic diagnosis | Diabetic foot  Venous leg ulcer  Pressure ulcer  Other (elaborate) |
| Comorbidities | Diabetes  Ischemic disease of the foot  Cancer  Other (elaborate) |
| Risk factors | BMI over 30  Smoking  Alcoholism  Corticosteroid treatment |
|  |  |
| **Initial characteristics of the wound** | |
| Localization | Lower extremity  Upper extremity  Torso/pelvis |
| Affected structures | Skin  subcutaneous tissue  muscle  tendon  joint  bone |
| Infection | None  biofilm  undermined wound bed  necrosis/gangrene |
| Exudate +/++/+++ | None  Serous  Bloody  Purulent |
| Pain | None  During dressing change  Occasional  Continuous |
| Malodor | None  Upon dressing removal  Through dressing  Intense |
| Microbiology | G+ / G+ / MRSA / Other (elaborate) |
| Wound size | cm x cm, depth in cm  *(size and volume were calculated from provided data)* |
|  |  |
| **Method of use of DebriEcaSan Alfa** | |
| Method of application |  |
| Exposure time |  |
| Frequency of dressing changes |  |
| Primary and secondary dressing |  |
| Compression |  |
| Other treatments |  |
| Antibiotics (number of days) |  |
|  |  |
| **Wound characteristics at week 3, 6, 9, and 12 (section repeats)** | |
| Affected structures | (see above) |
| Infection | (see above) |
| Exudate +/++/+++ | (see above) |
| Pain | (see above) |
| Malodor | (see above) |
| Microbiology | (see above) |
| Wound size | (see above) |
| Granulation | 0% - 25% - 50% - 75% - 100% |
| Epithelization | 0% - 25% - 50% - 75% - 100% |
| Complications | Free text |
| Tissue surrounding the wound | Healthy – erythema – maceration – other (elaborate) |
|  |  |
| **Comment, signature** | |
| Efficacy | Scale 1 (worst) to 10 (best) |
| Complications |  |
| Work with material |  |
| Patient comfort |  |
| Number of weeks till wound closure |  |
| Reason for discontinuation | Healed  Healing (discharged)  Still healing  Therapy change  Surgery  Other (elaborate) |
| Date |  |
| Consent |  |
| Signature |  |
