# Supplementary material for: The safety and efficacy of neutral electrolyzed water solution for wound irrigation: post-market clinical follow-up study
Source: Front Drug Saf Regul. 2025 Jan 16;4:1402684. doi: 10.3389/fdsfr.2024.1402684 (PMC12443096; doi:10.3389/fdsfr.2024.1402684)
Supplement: Supplementary file 2 [file Table8.docx]

Supplementary Material

## Supplementary Figure 8 – Wound healing

### Figure 8A – Wound granulation – development over time

### Figure 8B – Wound epithelization

### Figure 8C – Wound granulation over time

### Figure 8D – Wound epithelization over time
