# Supplementary material for: The safety and efficacy of neutral electrolyzed water solution for wound irrigation: post-market clinical follow-up study
Source: Front Drug Saf Regul. 2025 Jan 16;4:1402684. doi: 10.3389/fdsfr.2024.1402684 (PMC12443096; doi:10.3389/fdsfr.2024.1402684)
Supplement: Supplementary file 3 [file Table7.docx]

Supplementary Material

### Figure 7 – Wound malodor – development over time (number of patients and percentage)

|  | **Initial** | **%** | **3 W** | **%** | **6 W** | **%** | **9 W** | **%** | **12 W** | **%** |
| --- | --- | --- | --- | --- | --- | --- | --- | --- | --- | --- |
| Intense malodor | 37 | 16% | 6 | 3% | 1 | 0% | 0 | 0% | 0 | 0% |
| Malodor through dressing | 27 | 11% | 13 | 5% | 7 | 3% | 4 | 2% | 2 | 1% |
| Malodor upon dressing removal | 72 | 30% | 52 | 22% | 32 | 14% | 29 | 12% | 12 | 5% |
| No malodor | 101 | 43% | 166 | 70% | 197 | 83% | 204 | 86% | 223 | 94% |
