# Supplementary material for: The safety and efficacy of neutral electrolyzed water solution for wound irrigation: post-market clinical follow-up study
Source: Front Drug Saf Regul. 2025 Jan 16;4:1402684. doi: 10.3389/fdsfr.2024.1402684 (PMC12443096; doi:10.3389/fdsfr.2024.1402684)
Supplement: Supplementary file 4 [file Table9.docx]

Supplementary Material

## Supplementary Figure 9 – Wound healing: wound size and wound closure

### Figure 9A – Wound surface area size: development over time (graph)

### Figure 9B – Wound depth (graph)

### Figure 9C – Wound healing (graph)
