# Supplementary material for: The safety and efficacy of neutral electrolyzed water solution for wound irrigation: post-market clinical follow-up study
Source: Front Drug Saf Regul. 2025 Jan 16;4:1402684. doi: 10.3389/fdsfr.2024.1402684 (PMC12443096; doi:10.3389/fdsfr.2024.1402684)
Supplement: Supplementary file 5 [file Table2.docx]

Supplementary Material

## Supplementary Figure 2 – Characteristics of the treated population by age and sex

Of the 237 patients, 115 were male and 122 were female. More men were represented in the younger categories than women. This shift can be explained by a longer lifespan in women and an earlier onset of diabetes in men.

| **Study period 1/2019-12/2023** | | | | |
| --- | --- | --- | --- | --- |
|  | **Male** | **Male (%)** | **Female** | **Female (%)** |
| 45 years or under | 9 | 4% | 6 | 3% |
| 46 to 55 years | 19 | 8% | 9 | 4% |
| 56 to 65 years | 31 | 13% | 20 | 8% |
| 66 to 75 years | 38 | 16% | 47 | 20% |
| 76 to 85 years | 17 | 7% | 33 | 14% |
| 86 years and over | 1 | 0% | 7 | 3% |
