# Supplementary material for: The safety and efficacy of neutral electrolyzed water solution for wound irrigation: post-market clinical follow-up study
Source: Front Drug Saf Regul. 2025 Jan 16;4:1402684. doi: 10.3389/fdsfr.2024.1402684 (PMC12443096; doi:10.3389/fdsfr.2024.1402684)
Supplement: Supplementary file 6 [file Table5.docx]

Supplementary Material

## Supplementary Figure 5 – Wound healing

### Figure 5A – Symptoms of infection: biofilm, undermined wound bed, necrosis/gangrene (number of patients and percentage)

The number of wounds with infection symptoms steadily decreased over the 12 weeks of treatment.

|  | **Initial** | | **3 Weeks** | | **6 Weeks** | | **9 Weeks** | | **12 Weeks** | |
| --- | --- | --- | --- | --- | --- | --- | --- | --- | --- | --- |
| Biofilm | 69 | 29% | 118 | 50% | 95 | 40% | 72 | 30% | 36 | 15% |
| Undermined wound bed | 75 | 32% | 35 | 15% | 16 | 7% | 3 | 1% | 2 | 1% |
| Necrosis/gangrene | 58 | 24% | 19 | 8% | 5 | 2% | 3 | 1% | 0 | 0% |
| None | 35 | 15% | 65 | 27% | 121 | 51% | 159 | 67% | 199 | 84% |
| Total | 238* | 100% | 237 | 100% | 237 | 100% | 237 | 100% | 237 | 100% |

* 1 patient reported both necrosis/gangrene and undermined wound bed and is included twice.

### Figure 5B – Symptoms of infection: wound exudate (number of patients and percentage)

The number of wounds with purulent, and bloody exudate decreased over time, partly changing to serous exudate, before clearing up completely. The number of wounds with no exudate increased from 30 during the initial examination to 188 at week 12.

|  | **Initial** | **%** | **3W** | **%** | **6W** | **%** | **9W** | **%** | **12W** | **%** |
| --- | --- | --- | --- | --- | --- | --- | --- | --- | --- | --- |
| Serous exudate | 102 | 43% | 143 | 60% | 133 | 56% | 96 | 41% | 47 | 20% |
| Purulent exudate | 81 | 34% | 27 | 11% | 10 | 4% | 5 | 2% | 2 | 1% |
| Bloody exudate | 25 | 11% | 14 | 6% | 9 | 4% | 3 | 1% | 0 | 0% |
| No exudate | 30 | 13% | 53 | 22% | 85 | 36% | 133 | 56% | 188 | 79% |
| Exudate amount xxx | 14 | 6% | 5 | 2% | 3 | 1% | 1 | 0% | 1 | 0% |
| Exudate amount xx | 30 | 13% | 23 | 10% | 11 | 5% | 9 | 4% | 1 | 0% |
| Exudate amount x | 14 | 6% | 22 | 9% | 33 | 14% | 21 | 9% | 13 | 5% |
