# Supplementary material for: The safety and efficacy of neutral electrolyzed water solution for wound irrigation: post-market clinical follow-up study
Source: Front Drug Saf Regul. 2025 Jan 16;4:1402684. doi: 10.3389/fdsfr.2024.1402684 (PMC12443096; doi:10.3389/fdsfr.2024.1402684)
Supplement: Supplementary file 7 [file Table3.docx]

Supplementary Material

## Supplementary Figure 3 – Basic diagnosis, comorbidities and risk factors

### Figure 3A – Basic diagnosis by sex (Tabular representation)

|  | **Female** | **Female (%)** | **Male** | **Male (%)** | **Total** | **Total**  **(%)** |
| --- | --- | --- | --- | --- | --- | --- |
| venous leg ulcer | 45 | 19% | 46 | 19% | 91 | 38% |
| pressure ulcer | 23 | 10% | 18 | 8% | 41 | 17% |
| Diabetic foot | 12 | 5% | 16 | 7% | 28 | 12% |
| wound (traumatic) | 9 | 4% | 9 | 4% | 18 | 8% |
| wound (surgical) | 4 | 2% | 2 | 1% | 6 | 3% |
| wound (other) | 1 | 0% | 5 | 2% | 6 | 3% |
| wound dehiscence | 3 | 1% | 2 | 1% | 5 | 2% |
| radiation dermatitis (prophylaxis) | 3 | 1% |  | 0% | 3 | 1% |
| amputation | 3 | 1% |  | 0% | 3 | 1% |
| leg ulcer, combined ethiology | 1 | 0% | 1 | 0% | 2 | 1% |
| osteomyelitis | 1 | 0% | 1 | 0% | 2 | 1% |
| heel pressure ulcer |  | 0% | 2 | 1% | 2 | 1% |
| cancer (breast carcinoma) | 2 | 1% |  | 0% | 2 | 1% |
| wound (leg) |  | 0% | 2 | 1% | 2 | 1% |
| radiation dermatitis | 2 | 1% |  | 0% | 2 | 1% |
| stomic wound | 1 | 0% | 1 | 0% | 2 | 1% |
| wound (unspecified) | 2 | 1% |  | 0% | 2 | 1% |
| phlegmon |  | 0% | 2 | 1% | 2 | 1% |
| periproctal abscess, fistula |  | 0% | 1 | 0% | 1 | 0% |
| Erysipelas | 1 | 0% |  | 0% | 1 | 0% |
| fistula | 1 | 0% |  | 0% | 1 | 0% |
| blister |  | 0% | 1 | 0% | 1 | 0% |
| ischaemic foot ulcer |  | 0% | 1 | 0% | 1 | 0% |
| cancer (carcinoma of tongue) |  | 0% | 1 | 0% | 1 | 0% |
| wound of unknown etiology (susp. insect bite) | 1 | 0% |  | 0% | 1 | 0% |
| cancer (skin carcinoma) | 1 | 0% |  | 0% | 1 | 0% |
| open fracture |  | 0% | 1 | 0% | 1 | 0% |
| wound (scalp) | 1 | 0% |  | 0% | 1 | 0% |
| acute uremic syndrome | 1 | 0% |  | 0% | 1 | 0% |
| cancer (melanoma) | 1 | 0% |  | 0% | 1 | 0% |
| solar burn 2nd to 3rd degree |  | 0% | 1 | 0% | 1 | 0% |
| Burn, 3rd degree |  | 0% | 1 | 0% | 1 | 0% |
| vasculitis | 1 | 0% |  | 0% | 1 | 0% |
| Diabetic foot, defect after amputation of 2nd and 3rd toe | 1 | 0% |  | 0% | 1 | 0% |
| venous leg ulcer, wound post-plastic surgery | 1 | 0% |  | 0% | 1 | 0% |
| phlegmon/Erysipelas |  | 0% | 1 | 0% | 1 | 0% |

### Figure 3B – Comorbidities and risk factors (Tabular representation)

| **Comorbidity/risk factor** | **Male** | **Male (%)** | **Female** | **Female (%)** | **Total** | **Total (%)** |
| --- | --- | --- | --- | --- | --- | --- |
| BMI over 30 | 45 | 19% | 54 | 23% | 99 | 42% |
| Diabetes mellitus, incl. Insulin dependent | 48 | 20% | 42 | 18% | 90 | 38% |
| Periferal Artery Disease(PAD)/Ischemic foot/Critical Limb Ischemia (CLI) | 45 | 19% | 34 | 14% | 79 | 33% |
| smoking | 49 | 21% | 24 | 10% | 73 | 31% |
| Cancer | 12 | 5% | 15 | 6% | 27 | 11% |
| alcoholism | 16 | 7% | 6 | 3% | 22 | 9% |
| hypertension | 6 | 3% | 16 | 7% | 22 | 9% |
| None (no comorbidities, no risk factors) | 10 | 4% | 9 | 4% | 19 | 8% |
| corticosteroids | 3 | 1% | 9 | 4% | 12 | 5% |
| varicose veins | 3 | 1% | 4 | 2% | 7 | 3% |
| chronic venous insufficiency | 2 | 1% | 4 | 2% | 6 | 3% |
| COVID 19 | 0 | 0% | 5 | 2% | 5 | 2% |
| atrial fibrillation | 2 | 1% | 1 | 0% | 3 | 1% |
| lymphedema | 1 | 0% | 2 | 1% | 3 | 1% |
| polyneuropathy | 1 | 0% | 2 | 1% | 3 | 1% |
| anemia | 1 | 0% | 1 | 0% | 2 | 1% |
| cachexia | 1 | 0% | 1 | 0% | 2 | 1% |
| COPD | 0 | 0% | 2 | 1% | 2 | 1% |
| dialysis | 1 | 0% | 1 | 0% | 2 | 1% |
| hypothyreosis | 1 | 0% | 1 | 0% | 2 | 1% |
| kidney failure | 1 | 0% | 1 | 0% | 2 | 1% |
| neuropathy | 0 | 0% | 2 | 1% | 2 | 1% |
| peripheral artery disease | 1 | 0% | 1 | 0% | 2 | 1% |
| ischaemic heart disease | 1 | 0% | 1 | 0% | 2 | 1% |
| Status post-Covid | 0 | 0% | 2 | 1% | 2 | 1% |
| congestive right heart failure | 1 | 0% | 0 | 0% | 1 | 0% |
| pulmonary hypertension | 1 | 0% | 0 | 0% | 1 | 0% |
| alcoholic liver cirrhosis | 0 | 0% | 1 | 0% | 1 | 0% |
| arrhythmia | 1 | 0% | 0 | 0% | 1 | 0% |
| asthma | 0 | 0% | 1 | 0% | 1 | 0% |
| chronic kidney disease | 1 | 0% | 0 | 0% | 1 | 0% |
| dyspnea | 1 | 0% | 0 | 0% | 1 | 0% |
| heart failure | 0 | 0% | 1 | 0% | 1 | 0% |
| hyperkalemia | 1 | 0% | 0 | 0% | 1 | 0% |
| hyperlipoproteinemia | 0 | 0% | 1 | 0% | 1 | 0% |
| leg edema | 0 | 0% | 1 | 0% | 1 | 0% |
| multiple basaliomas | 0 | 0% | 1 | 0% | 1 | 0% |
| neutropenia | 1 | 0% | 0 | 0% | 1 | 0% |
| phlegmon | 1 | 0% | 0 | 0% | 1 | 0% |
| polymorbid | 0 | 0% | 1 | 0% | 1 | 0% |
| Prostate cancer | 1 | 0% | 0 | 0% | 1 | 0% |
| sacrum | 0 | 0% | 1 | 0% | 1 | 0% |
| scleroderma | 0 | 0% | 1 | 0% | 1 | 0% |
| status post serious car accident | 1 | 0% | 0 | 0% | 1 | 0% |
| Anoxic brain damage | 1 | 0% | 0 | 0% | 1 | 0% |
| anticoagulation with warfarin | 1 | 0% | 0 | 0% | 1 | 0% |
| casus socialis | 1 | 0% | 0 | 0% | 1 | 0% |
| celiac disease | 0 | 0% | 1 | 0% | 1 | 0% |
| chronic bronchitis | 1 | 0% | 0 | 0% | 1 | 0% |
| chronic cardiac insufficiency | 0 | 0% | 1 | 0% | 1 | 0% |
| Chronic heart failure | 0 | 0% | 1 | 0% | 1 | 0% |
| chronic renal insufficiency | 0 | 0% | 1 | 0% | 1 | 0% |
| coma | 0 | 0% | 1 | 0% | 1 | 0% |
| covid pneumonia | 1 | 0% | 0 | 0% | 1 | 0% |
| hemodialysis | 0 | 0% | 1 | 0% | 1 | 0% |
| Homelessness | 1 | 0% | 0 | 0% | 1 | 0% |
| immobilization syndrome | 0 | 0% | 1 | 0% | 1 | 0% |
| incontinence | 1 | 0% | 0 | 0% | 1 | 0% |
| malnutrition | 0 | 0% | 1 | 0% | 1 | 0% |
| paraplegia | 1 | 0% | 0 | 0% | 1 | 0% |
| Pseudoarthrosis tibiae congenita | 0 | 0% | 1 | 0% | 1 | 0% |
| psychiatric diagnosis | 1 | 0% | 0 | 0% | 1 | 0% |
| respiratory failure | 1 | 0% | 0 | 0% | 1 | 0% |
| Thyreoiditis | 0 | 0% | 1 | 0% | 1 | 0% |
