# Supplementary material for: The safety and efficacy of neutral electrolyzed water solution for wound irrigation: post-market clinical follow-up study
Source: Front Drug Saf Regul. 2025 Jan 16;4:1402684. doi: 10.3389/fdsfr.2024.1402684 (PMC12443096; doi:10.3389/fdsfr.2024.1402684)
Supplement: Supplementary file 8 [file Table4.docx]

Supplementary Material

### Figure 4 - The time lag between the injury and the first visit (graph)
