# Supplementary material for: The safety and efficacy of neutral electrolyzed water solution for wound irrigation: post-market clinical follow-up study
Source: Front Drug Saf Regul. 2025 Jan 16;4:1402684. doi: 10.3389/fdsfr.2024.1402684 (PMC12443096; doi:10.3389/fdsfr.2024.1402684)
Supplement: Supplementary file 9 [file Table11.docx]

Supplementary Material

## Supplementary Figure 11 – Antimicrobial properties of superoxidized solutions

Superoxidized solutions show efficacy against a number of aerobic and facultatively aerobic bacteria, anaerobic bacteria, viruses, bacterial spores, bacteriophages and Eukaryotes. They also show good efficacy against biofilms:

***Aerobic and facultatively aerobic bacteria:*** *Acinetobacter spp., Actinobacillus actinomycetemcomitans, Aeromonas liquefaciens, Alcaligenes faecalis, Bacillus subtilis, Bacillus cereus, Burkholderia cepacian, Citrobacter freundii, Campylobacter jejuni, Escherichia coli, Enterobacter aerogenes, Enterococcus spp., VRE, Flavobacter spp., Haemophilus influenzae, Helicobacter pylori, Lactobacillus spp, Legionella pneumophila, Listeria monocytogenes, Klebsiella spp., Micrococcus luteus, Mycobacterium spp., Proteus spp., Pseudomonas aeruginosa, Salmonella spp., Serratia marcescens, Staphylococcus spp., MRSA, MSSA, MRSE, Stentotrophomonas maltophilia, Streptococcus spp., and Xanthomonas maltophilia.*

***Anaerobic bacteria:*** *Actinomyces spp., Bifidobacterium bifidum, Bacteroides fragilis, Clostridium difficile, Eubacterium lentum, Fusobacterium nucleatum, Peptococcus niger, Peptostreptococcus anaerobius, Prevotella melaninogenica, Porphyromonas spp., Prevotella loeschii, Propionibacterium acnes, and Veillonella parvula.*

***Viruses:*** *FCV 2280, Flu A H1N1, Flu A H5N1, Flu A H9N2, Flu A H3N1, HIV 1, HSV 1, HSV 2, Norovirus, Polio 1, Rhino A1, RSV, WNV.*

***Bacterial Spores:*** *Bacillus anthracis, Bacillus atrophaeus, Bacillus cereus, Bacillus subtilis, Clostridium difficile, Clostridium perfringens, and Streptomyces spp.*

***Bacterophages:*** *Bacteriophage Qβ*

***Eukaryotes:*** *Aspergillus spp., Candida spp., Cryptosporidium parvum oocysts*

***Biofilms:*** *Staphylococcus aureus, Pseudomonas aeruginosa, Candida albicans, Enterococcus faecalis*
