# Supplementary material for: The safety and efficacy of neutral electrolyzed water solution for wound irrigation: post-market clinical follow-up study
Source: Front Drug Saf Regul. 2025 Jan 16;4:1402684. doi: 10.3389/fdsfr.2024.1402684 (PMC12443096; doi:10.3389/fdsfr.2024.1402684)
Supplement: Supplementary file 10 [file Table6.docx]

Supplementary Material

### Figure 6 – Reported pain over time

Pain reported by patients decreased in intensity and numbers. At the initial examination, 36 (15%) patients reported no pain. This number increased to 77 (32%) at week 3, 132 (56%) at week 6, 173 (73%) at week 9, and 209 (88%) at week 12.

|  | **Initial** | **%** | **3W** | **%** | **6W** | **%** | **9W** | **%** | **12W** | **%** |
| --- | --- | --- | --- | --- | --- | --- | --- | --- | --- | --- |
| Continuous pain | 51 | 22% | 13 | 5% | 4 | 2% | 2 | 1% | 1 | 0% |
| Pain during dressing change | 64 | 27% | 84 | 35% | 60 | 25% | 41 | 17% | 18 | 8% |
| Occasional pain | 86 | 36% | 63 | 27% | 41 | 17% | 21 | 9% | 9 | 4% |
| No pain reported | 36 | 15% | 77 | 32% | 132 | 56% | 173 | 73% | 209 | 88% |
