# Supplementary material for: The safety and efficacy of neutral electrolyzed water solution for wound irrigation: post-market clinical follow-up study
Source: Front Drug Saf Regul. 2025 Jan 16;4:1402684. doi: 10.3389/fdsfr.2024.1402684 (PMC12443096; doi:10.3389/fdsfr.2024.1402684)
Supplement: Supplementary file 11 [file Table10.docx]

Supplementary Material

**Figure 10 - State of the Art in the treatment of chronic wounds**

The table below provides a comprehensive summary of findings from scientific literature regarding the topical treatment of chronic wounds. No significant shift occurred in the treatment of diabetic foot ulcer, venous ulcer, pressure ulcers, and other types of chronic wounds.

| **Author/ year** | **Study type** | **Summary** | **SOS/HOCl** |
| --- | --- | --- | --- |
| O’Meara 2000 | SR of 30 studies (25 RCTs) | No existing evidence to support the use of systemic antimicrobial agents for chronic wound healing. Research into topical agents requires replication on a larger scale | Not specifically discussed |
| Peters 2012 | SR of 33 studies (29 RCTs) | No benefit has been reported with any other intervention, and, overall, there are currently no trial data to justify the adoption of any particular therapeutic approach in diabetic patients with infection of either soft tissue or bone of the foot. | In two studies, the use of *superoxidized water* was associated with a better outcome than soap or *povidone iodine*, but both had a high risk of bias |
| Eftekharizadeh 2016 | HTA (5 RCTs, 5 CTs, 1 rapid HTA, case series) | Most of these trials assessed similar sets of outcomes as the safety and effect on healing days to re-epithelization, healing rate, effect on infection bacterial counts and infection rates. SOS is a safe, effective and cost-effective irrigation and cleansing agent due to the performed analysis in comparison with current treatment as povidone iodine for treating wound infections | SOS is a safe, effective and cost-effective irrigation and cleansing agent compared to povidone iodine |
| Peters, 2016 | SR of 40 studies (37 RCTs and three cohort studies) | This SR revealed little evidence upon which to make recommendations for treatment of DFIs. There is a great need for further well-designed trials that will provide robust data upon which to make decisions about the most appropriate treatment of both skin and soft tissue infection and osteomyelitis in diabetic patients. | Four studies that compared the results of treating DFI with topical superoxidised water versus either soap or povidone iodine. No significant differences found. Weak evidence. |
| Dumville, 2017 | SR of 22 studies | Included studies employed various topical antimicrobial treatments, including antimicrobial dressings (e.g. silver, iodides), superoxidised aqueous solutions, zinc hyaluronate, silver sulphadiazine, tretinoin, pexiganan cream, and chloramine. The evidence of efficacy and safety of topical antimicrobial treatments for diabetic foot ulcers is limited by the availability of relatively few, mostly small, and poorly designed trials. | The evidence of effectiveness and safety of topical antimicrobial treatments, including SOS, for diabetic foot ulcers is limited. |
| Gold 2017 | Literature review, clinical recommendations based on expert panel consensus | a postprocedure regime using of hypochlorous acid, aimed at preventing infection, reducing inflammation, and promoting healing is proposed to have benefits over current regimes as it appears to be effective, safe, and well tolerated. | Hypochlorous acid has demonstrated efficacy and safety for pre- and postprocedure use. The safety of hypochlorous solution use demonstrated to be comparable to that of standard local antiseptics. |
| WHO 2017, 2021 | Application for the inclusion of electromysin to the list of essential medicines | The Panel’s recommendations were to cleanse the wound, followed by debridement, and to treat infected wounds with HOCl. In 2021, the WHO Expert Committee on Selection and Use of Essential Medicines reviewed the Application and ultimately decided not to add hypochlorous acid to the EML due to low quality of evidence. | The indications include diabetic foot ulcers, venous leg ulcers, pressure ulcers, postsurgical wounds, first-degree and second-degree burns and grafted and donor sites. |
| Kramer 2018 | Consensus on wound antisepsis | An *infected or critically colonized wound must be treated antiseptically*. In addition, systemic antibiotic therapy is required in case the infection spreads. If applied preventively, the *Wounds-at-Risk Score* allows an assessment of the risk for infection and thus appropriateness of the indication. | *Polyhexanide and hypochlorite are superior to PVP-iodine for the treatment of contaminated acute and chronic wounds*. |
| Khasim 2019 | Review of biomarkers in DFU and advanced therapies | All the advanced therapies and foot ulcer dressing materials are not suitable for all types of diabetic foot ulcers. The application of biocompatible nanoparticles holds a promising approach for designing dressing materials for the treatment of diabetic foot ulcer and reducing amputations | SOS is considered an advanced therapy. SOS is non toxic, having neutral pH water that contains ROS generated by the electrolysis of sodium chloride and water. SOS has antimicrobial activity against antibiotic resistant strains and it has been reported that this solution is effective and safe for treating infected DFU both in human and in the animal model. |
| Dayya 2022 | SR/MA/MR  10 SRs/6 SRs included in MA. 30 studies included. | Current published literature is unclear on which specific method of debridement ha beneficial effects and which has important public health and clinical implications, including amputation rates, complicating infection rates, QoL, wound healing and recurrence rates, time to complete healing, and costs. | SOS not specifically discussed |
| Garcia 2022 | SR – 11 clinical practice guidelines identified | A SR to compile and evaluate current recommendations in international clinical practice guidelines (CPGs) to develop more consistent clinical guidance. Silver sulfadiazine is the most recommended topical antimicrobial in low-resource settings. | ISBI Practice Guidelines for Burn Care, Part 2 recommends for deeper burn wounds (that are not expected to heal spontaneously) prior to surgical excision. |

*SR – systematic review; MA – meta-analysis; MR – meta-regression; RCT – randomized controlled trial; HTA – health technology assessment*
